# Regulation of Mammalian Autophagy by Class II and III PI 3-Kinases through PI3P Synthesis

**DOI:** 10.1371/journal.pone.0076405

**Published:** 2013-10-03

**Authors:** Kelly Devereaux, Claudia Dall’Armi, Abel Alcazar-Roman, Yuta Ogasawara, Xiang Zhou, Fan Wang, Akitsugu Yamamoto, Pietro De Camilli, Gilbert Di Paolo

**Affiliations:** 1 Department of Pathology and Cell Biology, Columbia University Medical Center, New York, New York, United States of America; 2 Taub Institute for Research on Alzheimer’s Disease and the Aging Brain, Columbia University Medical Center, New York, New York, United States of America; 3 Department of Cell Biology, Yale University School of Medicine, New Haven, Connecticut, United States of America; 4 Howard Hughes Medical Institute, Yale University School of Medicine, New Haven, Connecticut, United States of America; 5 Department of Animal Bio-Science, Faculty of Bio-Science, Nagahama Institute of Bio-Science and Technology, Nagahama, Shiga, Japan; 6 Department of Cell Biology, Duke University Medical Center, Durham, North Carolina, United States of America; 7 Department of Neurobiology, Duke University Medical Center, Durham, North Carolina, United States of America; University of Geveva, Switzerland

## Abstract

Synthesis of phosphatidylinositol-3-phosphate (PI3P) by Vps34, a class **III** phosphatidylinositol 3-kinase (PI3K), is critical for the initial steps of autophagosome (AP) biogenesis. Although Vps34 is the sole source of PI3P in budding yeast, mammalian cells can produce PI3P through alternate pathways, including direct synthesis by the class II PI3Ks; however, the physiological relevance of these alternate pathways in the context of autophagy is unknown. Here we generated *Vps34* knockout mouse embryonic fibroblasts (MEFs) and using a higher affinity 4x-FYVE finger PI3P-binding probe found a Vps34-independent pool of PI3P accounting for ^~^35% of the total amount of this lipid species by biochemical analysis. Importantly, WIPI-1, an autophagy-relevant PI3P probe, still formed some puncta upon starvation-induced autophagy in *Vps34* knockout MEFs. Additional characterization of autophagy by electron microscopy as well as protein degradation assays showed that while Vps34 is important for starvation-induced autophagy there is a significant component of functional autophagy occurring in the absence of Vps34. Given these findings, class II PI3Ks (α and β isoforms) were examined as potential positive regulators of autophagy. Depletion of class II PI3Ks reduced recruitment of WIPI-1 and LC3 to AP nucleation sites and caused an accumulation of the autophagy substrate, p62, which was exacerbated upon the concomitant ablation of Vps34. Our studies indicate that while Vps34 is the main PI3P source during autophagy, class II PI3Ks also significantly contribute to PI3P generation and regulate AP biogenesis.

## Introduction

Macroautophagy (hereafter referred to as autophagy) is a catabolic, homeostatic process that occurs at low, basal levels in all cells to ensure sufficient turnover of long-lived proteins and damaged organelles. Autophagy can be upregulated in response to nutrient deprivation and various types of stress, including oxidative stress, accumulation of misfolded proteins, bacterial and viral infection, in order to protect cells and promote their survival [1-5]. A key feature of autophagy is the biogenesis of autophagosomes (APs), which are large, double membrane vesicles that sequester cytoplasmic substrates and undergo a maturation process to ultimately fuse with lysosomes, allowing for the degradation of their cargoes. APs are formed via the nucleation and expansion of an isolation membrane cistern that elongates and seals around cytoplasmic components [1-6]. A series of autophagy-related (Atg) proteins as well as lipid signaling events together promote the remodeling intracellular membranes and regulate AP formation [7-10].

A critical signaling lipid involved in the control of autophagy is phosphatidlyinositol 3-phosphate (PI3P) [6,10-12]. During mammalian autophagy, spatially-restricted production of PI3P has been observed within subdomains of the endoplasmic reticulum (ER), termed “omegasomes” (for their omega-like shape), which become nucleation sites for AP biogenesis [13-15]. PI3P synthesis at these sites provides key localization cues to recruit effectors harboring PI3P-binding modules, such as the FYVE domain, PX domain and WD-repeat domains, to the isolation membrane. PI3P effectors, such as DFCP1 (FYVE domain) and Atg18/ WD repeat domain phosphoinositide-interacting (WIPI) protein family members (WD40 domains), promote AP biogenesis and operate as scaffold proteins that recruit Atgs required for downstream steps such as membrane elongation and closure [10-13,16]. Tight control of PI3P levels through the coordination of PI3Ks and PI3P phosphatases (e.g., Jumpy, MTMR3) at this site is critical in determining both size and production rate of APs [17,18]. Once the AP is formed, normal PI3P balance is also important for its maturation steps, including the trafficking of APs and their fusion with the endolysosomal organelles [8,10,11,19].

The best characterized pathway for PI3P synthesis involves the phosphorylation of PI on the 3’ position of the inositol ring by class III PI3K or Vps34 [20]. The lipid kinase activity of Vps34 was originally identified in budding yeast for its essential role in vacuolar hydrolase sorting from the late-Golgi to the vacuole [21,22] and was subsequently shown to be required for the autophagy pathway as well [8,10,11,13,23-31]. Later studies revealed that Vps34 operates in several, largely conserved complexes that determine its different physiological roles and regulate its lipid kinase activity [6,10,20,32]. In mammals, a core Vps34 complex consisting of Vps34, Beclin 1 (a homologue of yeast Vps30/Atg6), Vps15 and Atg14L is directed to the AP nucleation site along the ER membrane by Atg14L to promote local synthesis of PI3P and initiate AP biogenesis, likely at sites of contact between ER and mitochondria [11,13,27,29-31,33,34].

While Vps34 is the only source of PI3P and required for autophagy in budding yeast, whether this lipid kinase plays an essential role in mammalian autophagy has been an outstanding question in the field. Previous molecular genetic (e.g., siRNA) and pharmacological studies using standard PI3K inhibitors, such as wortmannin or 3-methyladenine (3MA), have suggested that Vps34 is critical for starvation-induced autophagy but Vps34-independent pathways for PI3P synthesis have yet to be ruled out [10,13,24,27,35]. Recently, several genetic mouse models have been generated to determine the role of mammalian Vps34 in autophagy and other physiological processes; however, conflicting conclusions have been drawn concerning its role in autophagy in particular. For instance, studies in various conditional knockout (KO) mouse models have reported a spectrum of findings, ranging from no autophagy phenotypes in sensory neurons [36] and T-lymphocytes [37] to severe autophagy defects in the latter cell type [38,39], fibroblasts, cardiomyocytes, hepatocytes [40], podocytes [41] and lens cells [42]. The answer to whether Vps34 plays an essential role in mammalian autophagy is more complex than originally anticipated. In fact, differences in autophagy requirements may not only reflect cell type, but also genetic background, cell state and type of autophagy-inducing stimuli, continuing to raise the question of whether sources of PI3P besides Vps34 can regulate autophagy.

In addition to Vps34, the class II PI3Ks catalyze the phosphorylation of PI to produce PI3P and a class II isoform, *pi3c2α*, was recently identified as a "hit" in a large autophagy interaction network screen, making this family of PI3Ks an obvious candidate PI3P source to test [43]. In this study, we generated *Vps34* KO MEFs from the Zhang et al mouse model and observed both PI3P production and a significant amount of functional APs forming independently of Vps34. Using an siRNA knockdown approach, we found that the class II PI3Ks also synthesize PI3P to promote autophagy. Our results confirm the notion that Vps34 is clearly important for autophagy, but also demonstrate that functional autophagy still occurs independently of Vps34 and that the class II PI3Ks are positive regulators of this process.

## Results

### Characterization of *Vps34* KO MEFs and their autophagy machinery

**Figure 1:**
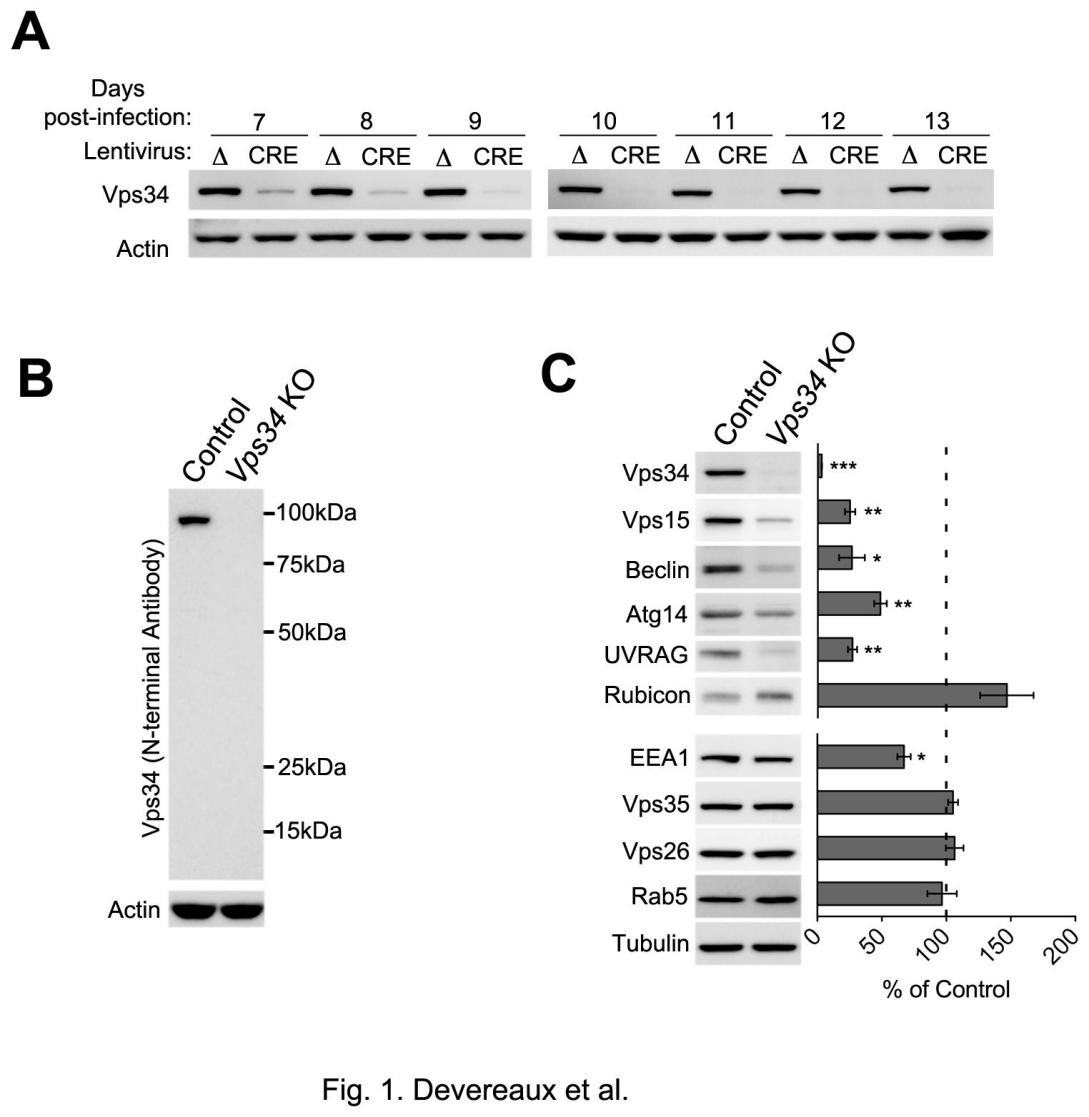
Ablation of Vps34 alters levels of Vps34 complex proteins. (**A**) Western blot analysis showing a time-course of Vps34 protein levels in *Vps34* ^Flox/Flox^ MEFs infected with lentiviruses expressing either an inactive Cre (Δ) or active (CRE) full-length Cre recombinase for 7-13 days. (**B**) Western blot using an antibody directed to the NH_2_-terminus of Vps34 in control and *Vps34* KO cell extracts. (**C**) Western blot analysis of Vps34 complex components and endosomal protein levels in control and *Vps34* KO MEF lysates. Quantification of protein levels after normalization to tubulin (n=3).

To investigate the contribution of Vps34 to starvation-induced autophagy, *Vps34* ^Flox/Flox^ MEFs were generated and characterized. *Vps34* ^Flox/Flox^ MEFs were immortalized by multiple passages and infected with lentiviruses expressing either a catalytically-inactive or active, nuclear Cre recombinase fused to eGFP (green) or tdTomato fluorescent protein (red) to generate control and *Vps34* KO cells, respectively (see Materials and Methods). Expression of active Cre, but not inactive Cre, resulted in the progressive loss of Vps34 immunoreactivity by Western blot analyses over the course of several days with complete loss of expression reproducibly achieved by 9 to 10 days (Figure 1A, S1*A*). Importantly, although exons 17 and 18 were targeted, antibodies directed to the very NH_2_-terminus of Vps34 (i.e., amino acids 1-40) did not reveal any truncated fragments of Vps34 at day 10 post-infection (Figure 1B). This finding together with the original analysis of *Vps34* mRNA in Cre-expressing *Vps34* ^Flox/Flox^ tissue [36] confirms that this targeting strategy successfully produces a *null* mutant.

Membranous organelles were examined and no major alterations of the ER, Golgi complex and lysosomes were found at day 10 post-infection based on the immunofluorescent analysis of protein disulfide isomerase (PDI), giantin, and LAMP1, respectively (Figure S1B). There was, however, an enlargement of the EEA1-positive early endosomal compartment, consistent with other studies [36,44-46] (Figure S1B). Notably, prolonged ablation of Vps34 for an additional 4-5 days (day 14-15 post-infection) consistently resulted in cell vacuolization (Figure S1C), a breakdown of the lysosomal compartment (as evidenced by an aberrant LAMP1 compartment) (Figure S1C) and impaired lysosomal degradation of the EGF receptor in response to EGF stimulation (Figure S1D). These features, which have been reported elsewhere [40,45], reflect the reduced viability resulting from the chronic loss of Vps34, a factor potentially confounding the analysis of the physiological function of Vps34 in autophagy. We thus focused our analysis at 10 days post-infection prior to the breakdown of the endolysosomal system.

Next, we examined levels of relevant autophagy and endosomal proteins. Ablation of Vps34 resulted in a significant decrease in levels of Vps34 complex members, including Vps15/p150 and Beclin 1, as previously reported [38], as well as Atg14L and UVRAG. In contrast, Rubicon, a negative regulator of Vps34 [29,30], showed increased levels in the KO. Levels of proteins operating in the endosomal pathway were also assessed and no changes were found in the expression levels of the retromer subunits Vps26 and Vps35 or Rab5, although EEA1 levels were slightly decreased (Figure 1C). These data indicate that Vps34 ablation results in the destabilization of molecular components previously reported to associate with Vps34 complexes as well as the endosomal PI3P effector, EEA1.

### Steady-state and starvation-induced PI3P detected in *Vps34* KO MEFs

To quantitatively determine the amount of PI3P produced by Vps34, phosphoinositides were measured in control and *Vps34* KO MEFs. For such large scale biochemical studies, control and *Vps34* KO cells were generated from *Vps34* ^Flox/Flox^ Cre-ER MEFs (see Materials and Methods). Specifically, cells were radiolabeled with [^3^H] *myo*-inositol, high performance liquid chromatography (HPLC) was performed and phosphoinositides levels were detected by radioactivity. We found a ^~^65% decrease in PI3P levels in *Vps34* KO MEFs relative to controls and no obvious alterations in other inositol lipids levels detected by the assay, such as PI, PI4P and PI(4,5)P_2_. Therefore, we conclude that in our fibroblasts, Vps34 accounts for the majority of cellular PI3P levels and that ^~^35% of this lipid can be synthesized through alternate pathways.

**Figure 2:**
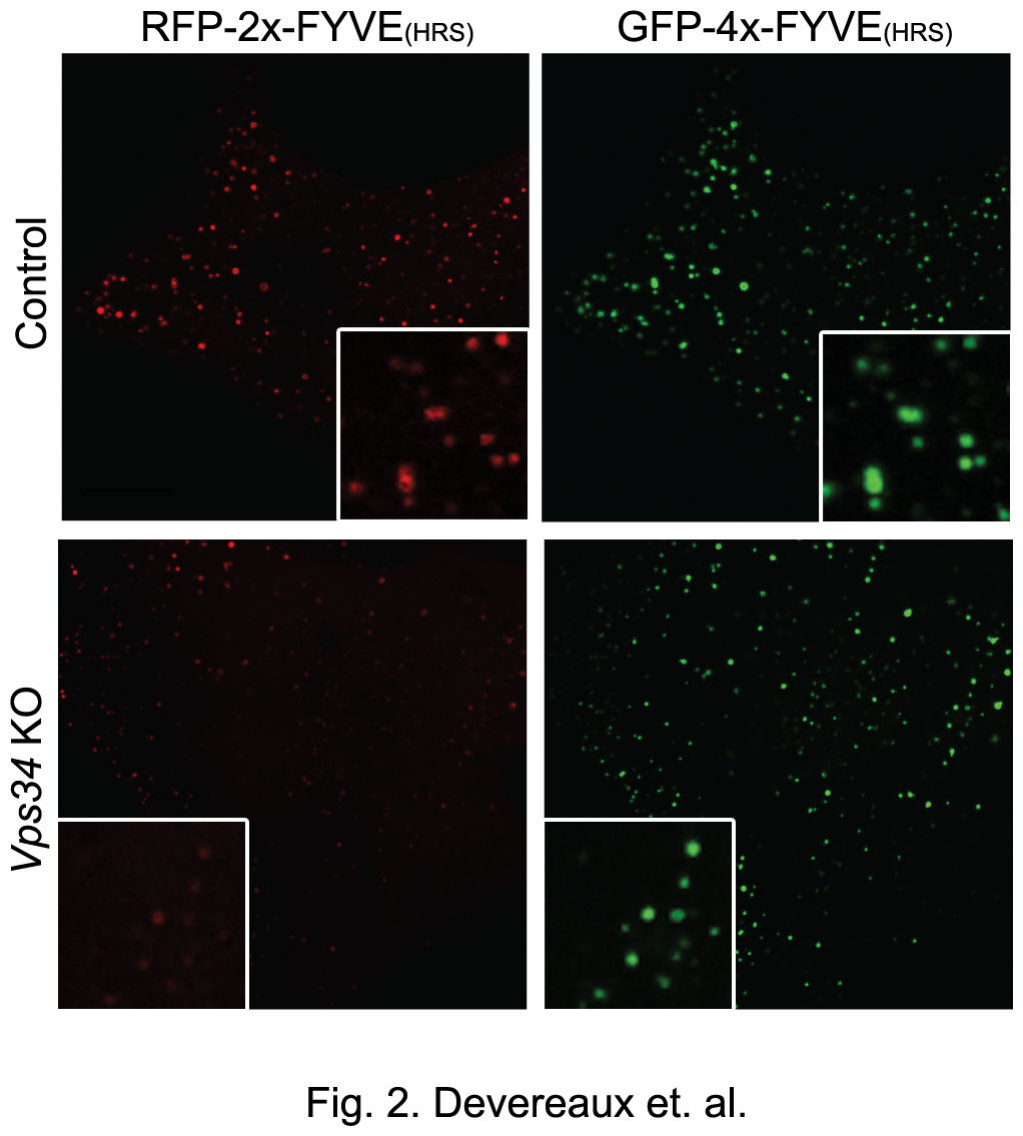
A higher affinity PI3P-binding probe, 4x-FYVE^Hrs^, reveals a larger pool of intracellular PI3P in the absence of Vps34 compared to the conventional 2x-FYVE^Hrs^ probe. Control and *Vps34* KO MEFs were transiently transfected with both RFP-2x-FYVE^Hrs^ and GFP-4x-FYVE^Hrs^ PI3P-binding constructs for 24hr, grown in normal media and fixed. Confocal microscopy analysis of RFP-2x-FYVE^Hrs^ (red) and GFP-4x-FYVE^Hrs^ (green) is shown. Scale bar: 10 µm.

Additionally, we detected intracellular PI3P using fluorescence microscopy. Historically, the detection and localization of intracellular PI3P has relied on the use of the FYVE domain (i.e., a zinc finger originally identified in **F**ab1, **Y**OTB, **V**ac1p, EEA1) as a genetically-encoded or purified PI3P-binding probe [47-49]). The first of these biosensors consisted of a tandem FYVE domain from Hrs (2xFYVE^Hrs^) which showed PI3P concentrated on the limiting and intraluminal membranes of endosomes [50]. Here control and *Vps34* KO MEFs were transiently transfected with RFP-2x-FYVE^Hrs^. In control MEFs, RFP-2x-FYVE^Hrs^ localized to vesicular structures, consistent with previous studies showing PI3P on endosomes [50]. In contrast, the RFP-2x-FYVE^Hrs^ fluorescent signal was largely diffuse in *Vps34* KO MEFs, which is also observed upon treatment with wortmannin and in MEFs where Vps15, the lipid kinase regulator of Vps34, is knocked out [50,51]. In order to improve the sensitivity and threshold of PI3P detection knowing that other pathways synthesize ~35% of the total PI3P, a high affinity 4xFYVE^Hrs^ probe was generated, based on the work of others suggesting that combining multiple FYVE domains may enhance PI3P detection or sequestration through an avidity effect [52]. In fact, transfection with the GFP-4x-FYVE^Hrs^ probe produced a more robust signal and revealed a pool of PI3P underestimated by the traditional 2x-FYVE probes in *Vps34* KO MEFs. Importantly, the 4x-FYVE^Hrs^ clearly confirmed PI3P synthesis occurs via other pathways (Figure 2).

**Figure 3:**
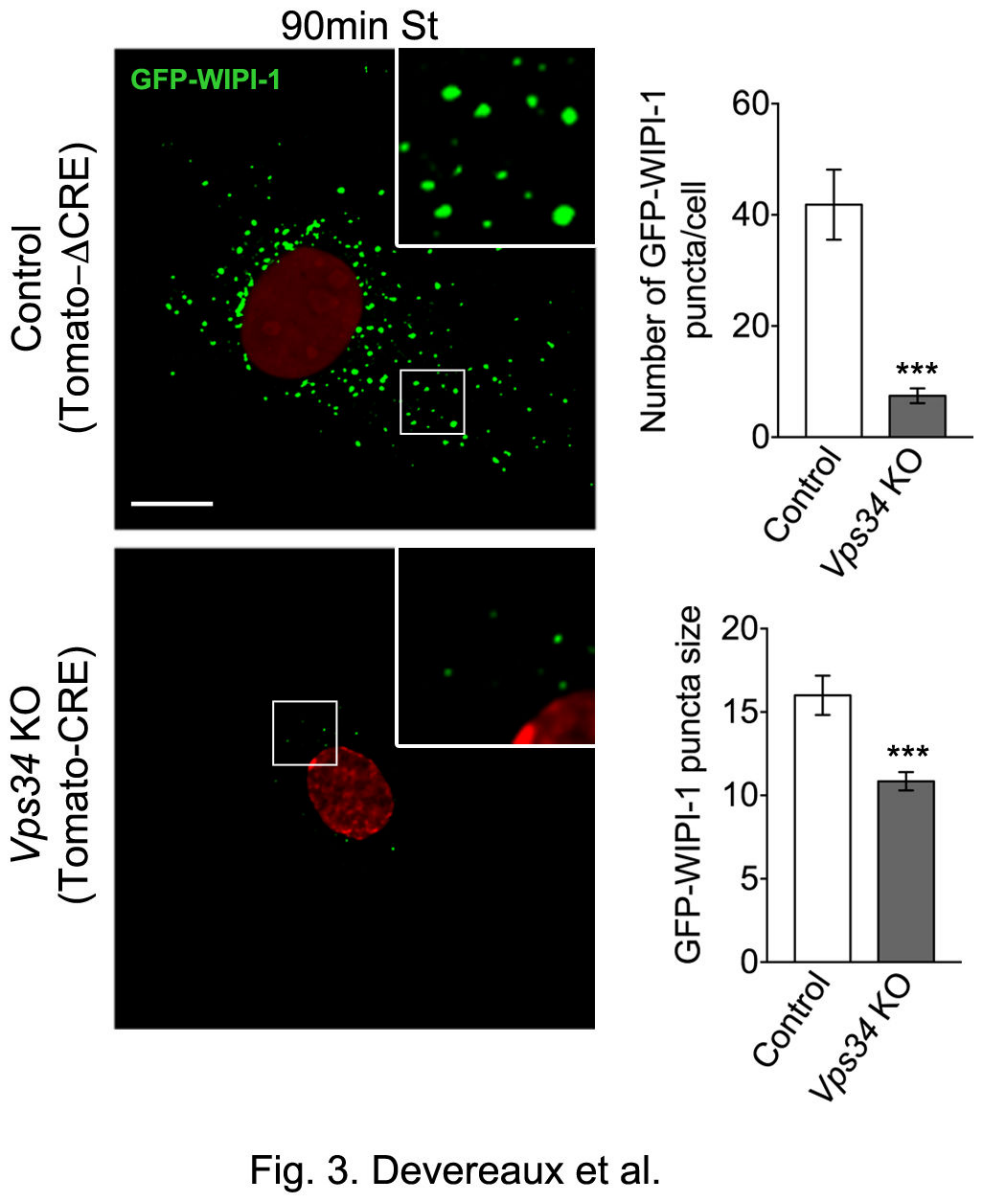
Recruitment of WIPI-1, a PI3P-binding protein, to sites of AP biogenesis occurs in *Vps34* KO MEFs but at diminished levels. Control and *Vps34* KO MEFs were transiently transfected with GFP-WIPI-1 for 24 hrs, cultured in HBSS for 90 min (90 min St) and fixed. Left: Confocal microscopy analysis of GFP-WIPI-1 fluorescence (green). Nuclear inactive and active Tomato-Cre is shown in red. The contrast was enhanced to reveal the WIPI puncta over the cytosolic background fluorescence. Right: Quantification of the number of GFP-WIPI-1 puncta (n=13-15 cells). Scale bar: 10 µm.

Next, we wanted to assess whether PI3P synthesis still occurred during starvation-induced autophagy in *Vps34* KO MEFs. As mentioned previously, PI3P plays an important role in recruiting PI3P effectors to the site of AP biogenesis. WD repeat domain phosphoinositide-interacting proteins (WIPI-1 and 2), mammalian orthologues of yeast Atg18, are a major family of PI3P-binding effectors that localize to the site of nucleation and promote AP formation; therefore, they are often used to monitor PI3P synthesis during autophagy [16,53]. Here, GFP-WIPI-1 was transiently transfected in control and *Vps34* KO MEFs and its localization examined under normal media and starvation conditions (HBSS for 90min). As previously observed, GFP-WIPI-1 remained largely soluble under normal media, basal autophagy conditions (data not shown) and formed punctate structures in control cells during starvation [53] (Figure 3). In *Vps34* KO MEFs, an ^~^80% and ^~^30% reduction in the number and size of GFP-WIPI-1 puncta was observed during starvation compared to control MEFs, respectively (Figure 3). Importantly, however, some WIPI-1 recruitment still occurred in the absence of Vps34, suggesting that PI3P production during autophagy may occur through other synthesis pathways, such as the class II PI3Ks.

### Autophagosome formation is reduced, but not abolished in *Vps34* KO MEFs

**Figure 4:**
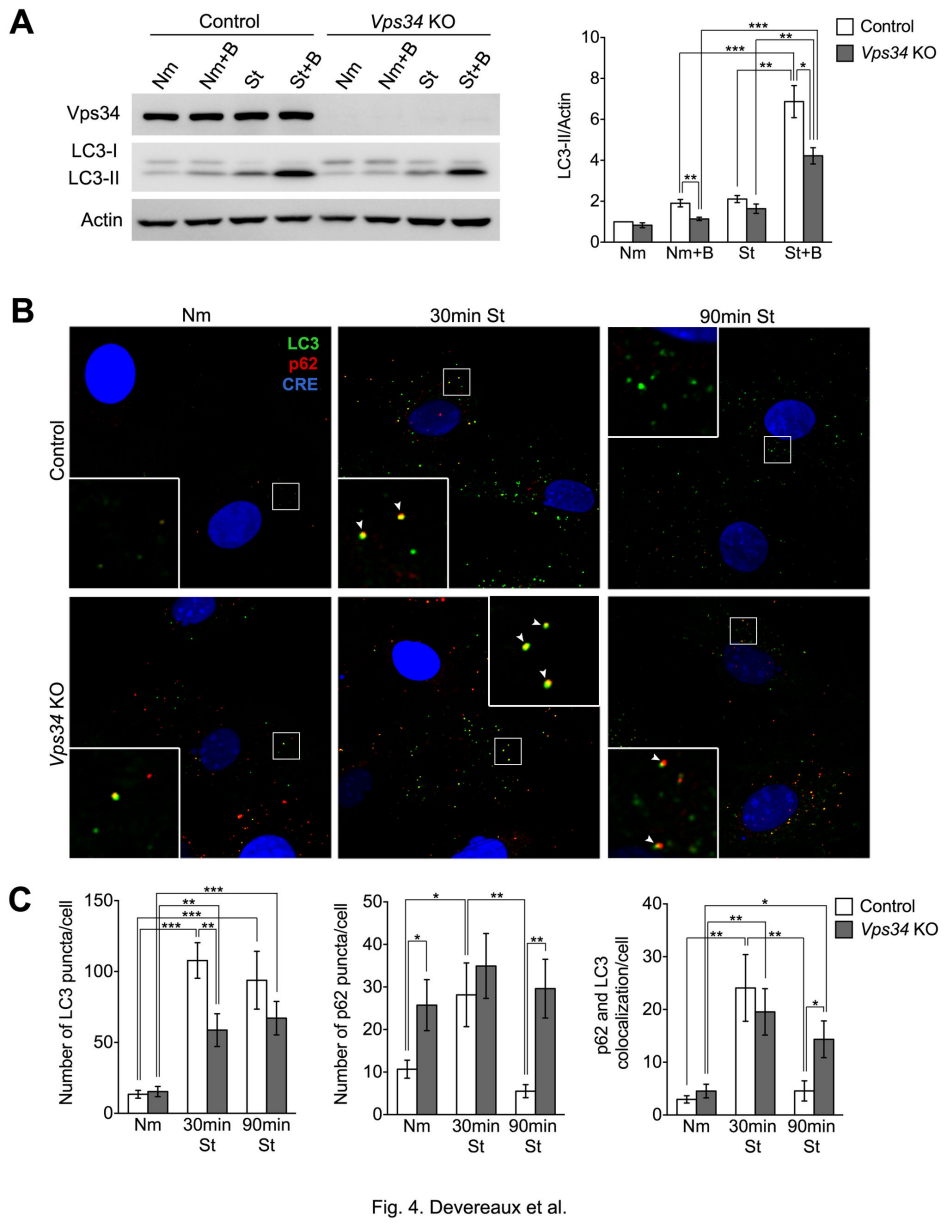
*Vps34* KO MEFs show a decrease in LC3 conjugation and LC3 puncta formation upon starvation. (**A**) Control and *Vps34* KO MEFs were cultured in normal medium (N) or HBSS (St) in the presence or absence of 50nM Bafilomycin (Nm+B or St+B, respectively) for 90 min. Right: Lysates were analyzed by immunoblotting using the indicated antibodies. Left: Relative LC3-II levels normalized to actin (n=4). (**B**) Control and *Vps34* KO MEFs were cultured in normal media (Nm) or HBSS (St) for 30 and 90 min, fixed and immunostained. Confocal analysis of LC3, p62 and GFP-Cre fluorescence, which is artificially shown in green, red and blue colors, respectively. Arrowheads indicate LC3 and p62 colocalization (yellow). Scale bar: 10 µm. (**C**) Quantification of the number of LC3 (left panel) and p62 puncta (middle panel) per cell. Colocalization of p62 with LC3 puncta is also shown (right panel) (colocalization was defined as the number of pixels overlapping in the p62 and LC3 channels normalized per cell) (n=35-45 cells).

We next investigated the ability of Vps34-deficient cells to form and clear APs in response to nutrient deprivation by measuring LC3 lipidation. Activation of autophagy results in the conversion of LC3-I to its lipidated form LC3-II, a process that can be monitored by Western blot analysis. Experiments are typically performed under normal media and starvation conditions in the presence or absence of proton pump blocker Bafilomycin A1 (Baf), which blocks the acidification of lysosomes and thus the clearance of APs and enables the assessment of autophagic flux. Levels of LC3-II were similarly low in both control and *Vps34* KO cells under basal conditions (Nm), although more LC3-II was apparent in control cells after the addition of Baf (Nm+B), suggesting a slightly higher rate of basal autophagy. While LC3-II levels remained low with no genotype-specific differences after nutrient deprivation (St) for 90 min, changes in autophagic flux were observed after the addition of Baf into the starvation media (St+B). Specifically, a more robust increase in the levels of LC3-II was seen in control cells (^~^3.3-fold) compared to *Vps34* KO cells (^~^2.5-fold), suggesting a decline in the rate of AP biogenesis in the absence of Vps34 in response to starvation (Figure 4A). This result suggests that while Vps34 may be an important modulator of autophagy, it is not essential for starvation-induced autophagy.

Next, endogenous LC3 localization was examined by confocal microscopy during starvation-induced autophagy. Under normal media conditions, LC3 immunofluorescence displayed a predominantly diffuse distribution with few basal LC3-positive compartments in control and Vps34-deficient cells. After 30 min starvation, LC3 was recruited to APs and an ~8-fold increase in the average number of fluorescent LC3 puncta was observed in control MEFs, whereas a less robust, ~4-fold increase was observed in *Vps34* KO MEFs. A more prolonged, 90 min starvation produced comparable results (Figure 4B,C). Levels of p62, an AP cargo, as well as its relationship with the LC3 compartment were also examined by immunofluorescence. In control MEFs, the baseline number of p62 puncta was low under normal media conditions, transiently increased by ~2.5-fold after 30 min of starvation, but then returned back to basal levels by 90 min. Likewise, the amount of colocalization between LC3 and p62 increased at 30 min and then returned to basal levels at 90 min as clearance of p62 by APs occurred over time. In contrast, the number of p62 puncta was 2.5-fold higher in *Vps34* KO MEFs under normal media compared to control cells and remained largely unaffected by starvation. Although the colocalization between p62 and LC3 significantly increased in the *Vps34* KO cells after 30 min starvation, the higher colocalization persisted after 90 min (Figure 4B,C). Altogether, our results suggest that LC3 can still be mobilized in response to autophagy stimulation in Vps34-deficient MEFs, but not as efficiently as in control MEFs. Also, given the persistent increase in p62 observed in the *Vps34* KO MEFs, it seems that although autophagy may be occurring in the absence of Vps34, levels are suboptimal for the efficient clearance of this cargo.

**Figure 5:**
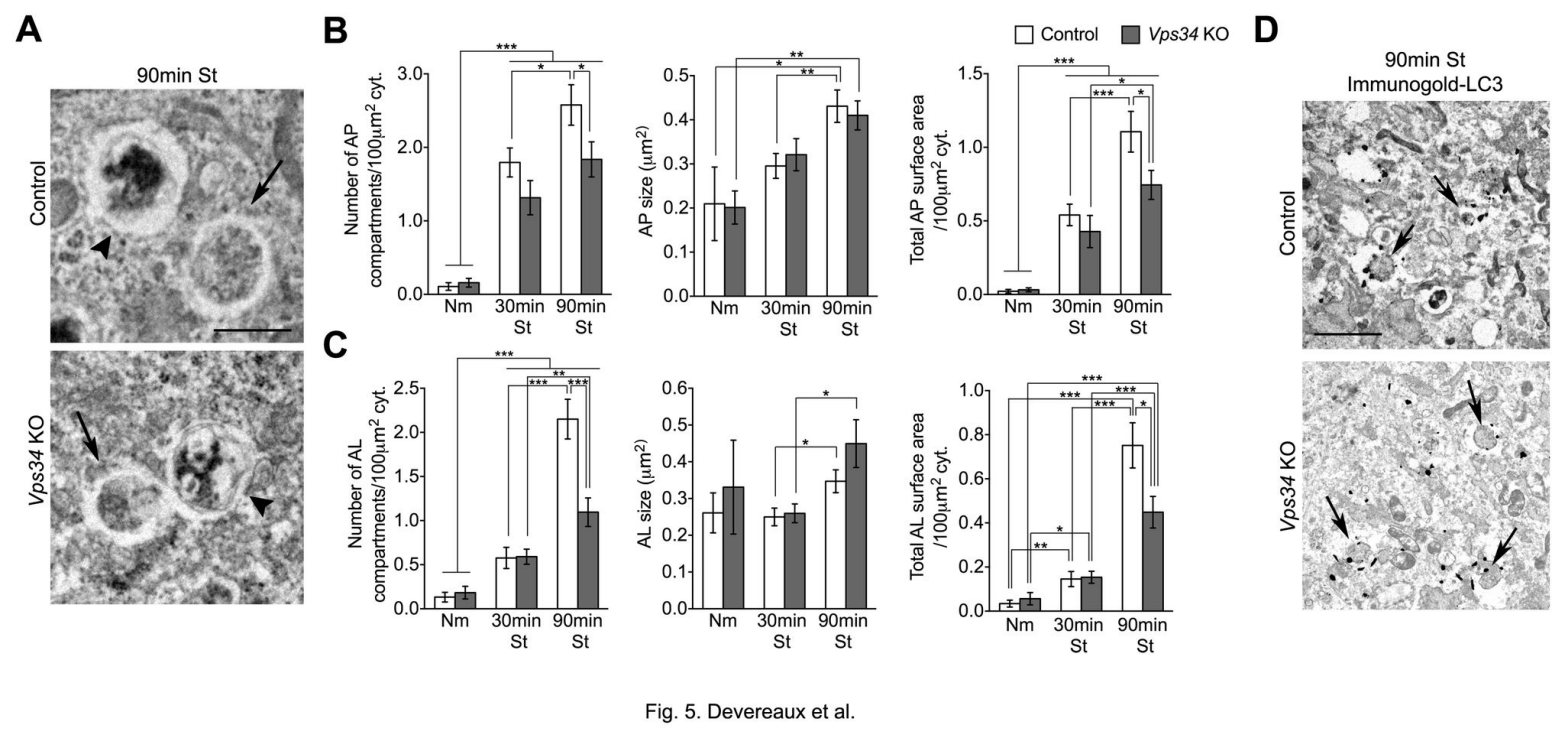
Lack of Vps34 decreases, but does not abolish the formation of autophagosomes and autophagolysosomes upon starvation. (**A**) Electron micrographs of control and *Vps34* KO MEFs cultured in normal media or after 30 and 90 min (shown) of HBSS starvation. Arrows, autophagosomes. Arrowheads, autophagolysosomes. Scale bar: 1 µm. (**B**) Quantification of average number of autophagosomes (AP), average size (µm^2^) of APs and the total AP surface area/100μm^2^ of cytoplasm. (**C**) Quantification of average number of autophagolysosomes (AL), average size (µm^2^) of ALs and the total AL surface area/100μm^2^ of cytoplasm. In both AP and AL quantifications, 30 cells were analyzed. (**D**) Immuno-gold electron microscopic analysis of endogenous LC3 in control and *Vps34* KO MEFs. Arrows, LC3 immunoreactive APs. Scale bar: 1 µm.

To more quantitatively compare APs and autophagolysosomes (ALs) in control and Vps34-deficient cells, standard electron microscopy (EM) was performed. Under basal conditions, APs and ALs were rarely observed, and no genotype-specific differences were found (Fig. 5A-C). Starving cells for 30 min caused an increase both in the number and total surface area covered by APs and ALs with no genotype-specific differences. The number and surface area of APs and ALs further increased after 90 min, but not as robustly in *Vps34* KO MEFs. The average size of APs and ALs also continued to increase with prolonged starvation, but was not affected by the genotype (Fig. 5B,C). Finally, immunogold-EM using an anti-LC3 revealed that APs observed upon starvation in Vps34-deficient cells are decorated with gold particles indistinguishably from control cells (Figure 5D), indicating that LC3 is normally recruited to the AP membranes in the absence of Vps34. These data confirm that ablation of Vps34 does not prevent the formation of APs and ALs, but does result in the generation of fewer autophagic vacuoles in response to nutrient deprivation, consistent with the light microscopic observations.

### Vps34 mediates approximately half of autophagic protein degradation

**Figure 6:**
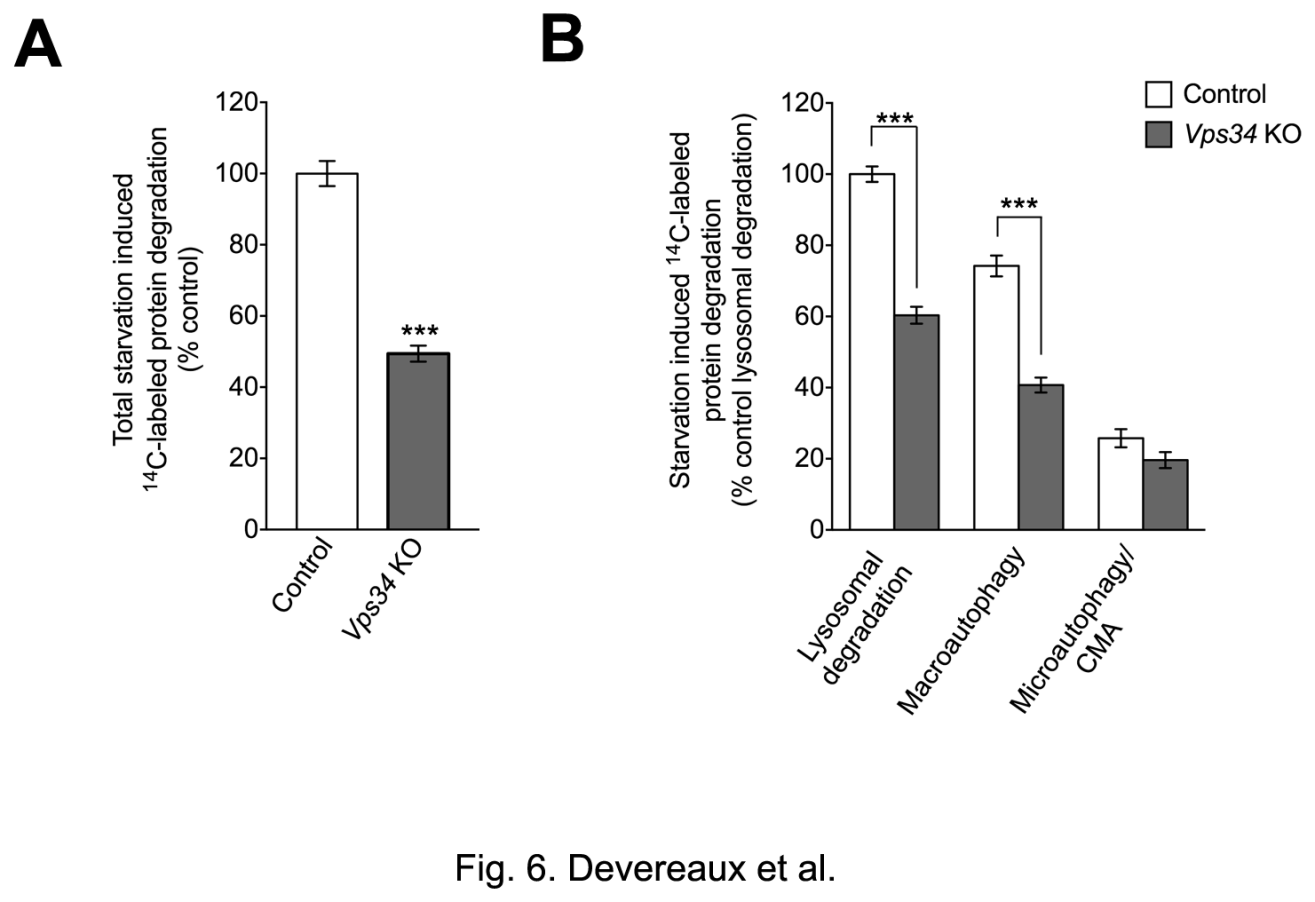
Macroautophagy-mediated protein degradation is partially impaired in *Vps34* null MEFs. (**A**) Quantification of total [^14^C]-valine long-lived protein degradation induced by nutrient deprivation (HBSS) in control and *Vps34* KO MEFs. (**B**) Assessment of autophagy efficiency in control and *Vps34* KO MEFs by ^14^C-valine long-lived protein degradation under starvation (HBSS), starvation with 3MA or starvation with NH_4_Cl conditions (see Methods) (n=8 for both A and B).

Given that LC3 conjugation and APs and ALs were still observed in *Vps34* KO cells, it was important to assess the functionality of the remaining autophagy. A [^14^C]-valine-labeled long-lived protein assay was performed in the presence of PI3K inhibitors (3MA or wortmannin) to inhibit autophagy, or the weak base ammonium chloride to inhibit lysosomal degradation, allowing us to determine the contribution of (macro)autophagy to the overall lysosomal degradation (see Materials and Methods). Since 3MA and wortmannin are generally thought to block autophagy by targeting Vps34, it was first necessary to determine whether this was, in fact, the case. Both 3MA and wortmannin dramatically reduced the lipidation of LC3 during starvation in both control and *Vps34* KO MEFs in the presence and absence of Baf (Figure S3A,B). This finding demonstrated that these drugs have additional autophagy-relevant targets besides Vps34 and can, therefore, still can be used to effectively block (macro)autophagy in *Vps34* KO MEFs. Here, we observed a 50% decrease in total starvation-induced proteolysis in *Vps34* KO MEFs by protein degradation assay (Figure 6A). Importantly, the 3MA-inhibitable component of lysosomal protein degradation, which corresponds to (macro)autophagy, was reduced by ^~^50% in Vps34-deficient MEFs upon nutrient deprivation, accounting for this significant decrease in total lysosomal protein degradation (Figure 6B). In contrast, Vps34 ablation only caused a decreasing trend in protein degradation through the microautophagy and/or chaperone-mediated autophagy pathways, suggesting that these lysosomal functions are not affected by the lack of Vps34 at this time-point of study (Figure 6B). Therefore, the reduction in autophagy function upon acute deletion of Vps34, most likely reflects a decrease in overall autophagosome number at Day 10 rather than impaired autophagosome clearance. However, prolonged ablation of Vps34 results in the progressive accumulation of p62 by Western blot analysis and immunofluorescence, possibly due to both reduced autophagy efficacy and a progressive decline in lysosomal function (Figure S2).

### Class II PI3Ks contribute a pool of PI3P in MEFs

**Figure 7:**
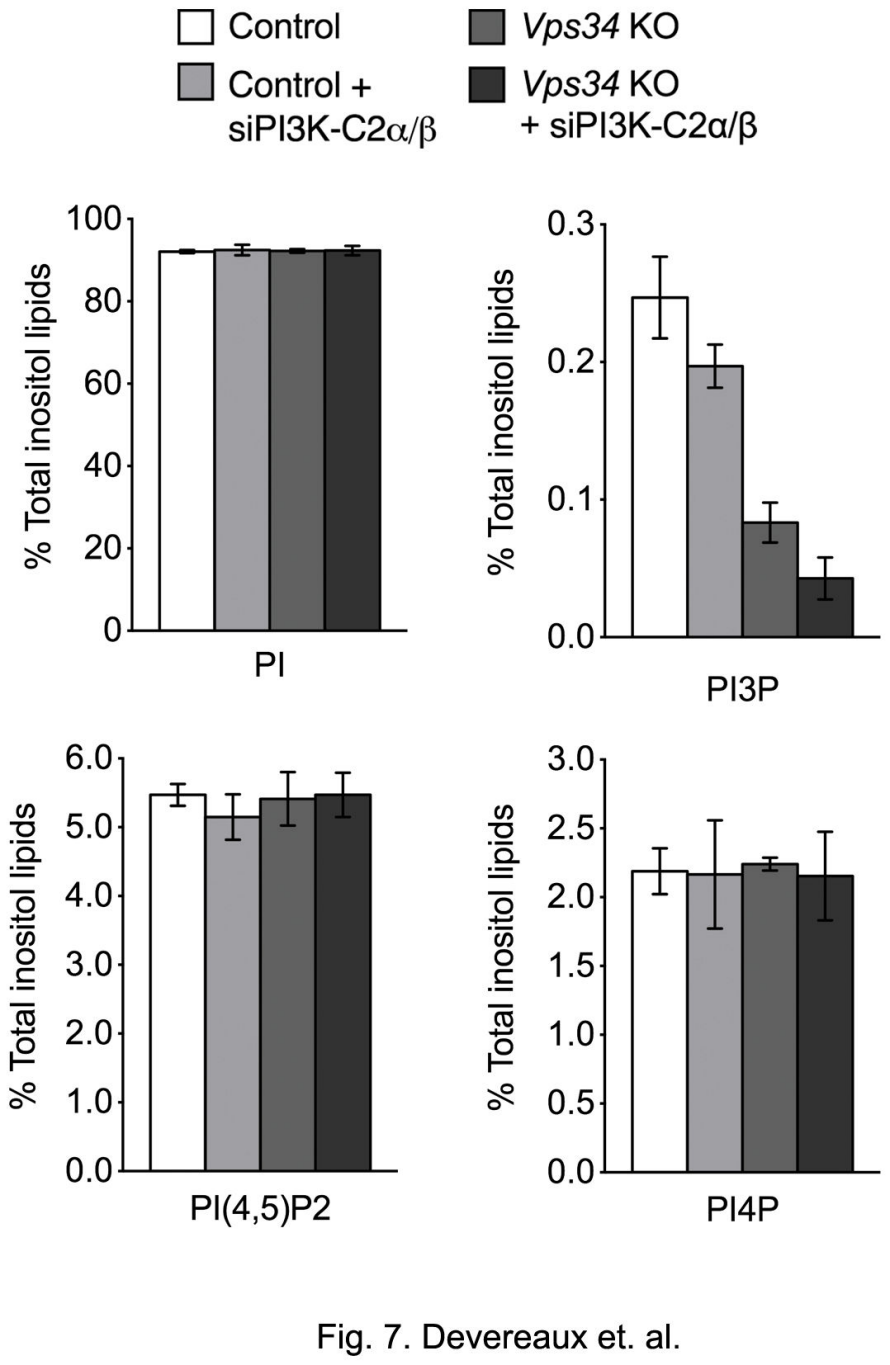
Quantification of Vps34-dependent and independent sources of PI3P in MEFs. *Vps34* KO MEFs alone or transfected with mock or PI3K-C2α/β siRNA for 48 hrs grown in normal medium supplemented with [^3^H] *myo*-inositol. [^3^H]-labeled phosphoinositides were extracted, deacylated and analyzed by HPLC and scintillation detection. Graph indicates relative levels of phosphatidylinositol (PI), phosphatidylinositol 3-phosphate (PI3P), phosphatidylinositol 4-phosphate (PI4P), phosphatidylinositol 4,5-bisphosphate (PI(4,5)P2), as a percentage of total inositol lipid abundance (n=3).

After establishing that functional autophagy still occurs in *Vps34* KO MEFs, we determined the contribution of PI3P synthesis by class II PI3Ks. Phosphoinositide levels were measured as described above (see Materials and Methods). Control and *Vps34* KO cells were treated with mock siRNA or both PI3K-C2α and β siRNAs (the third isoform, PI3K-C2γ, appears to be mostly expressed in the liver [54]). PI3K-C2α and β expression was consistently abolished after 48hrs of PI3K-C2α/β siRNA treatment with no major impact on Vps34 levels (Figure S4). Interestingly, silencing of class II PI3Ks in control and *Vps34* KO cells caused a comparable relative decrease in the overall levels of PI3P. Specifically, we observed a ^~^20% decrease in PI3P levels in control cells treated with PI3K-C2α/β siRNA as compared to mock siRNA (Figure 7). Likewise, an additional ^~^15% reduction in PI3P levels was observed when the class II PI3Ks were silenced in the *Vps34* KO background (Figure 7). Taken together, we conclude that the contributions of Vps34 and class II PI3Ks to total PI3P levels are additive and non-compensatory. Additionally, we observed that approximately half of the amount of PI3P produced in *Vps34* KO MEFs is accounted for by class II PI3Ks.

### Class II PI3Ks positively regulate autophagy

**Figure 8:**
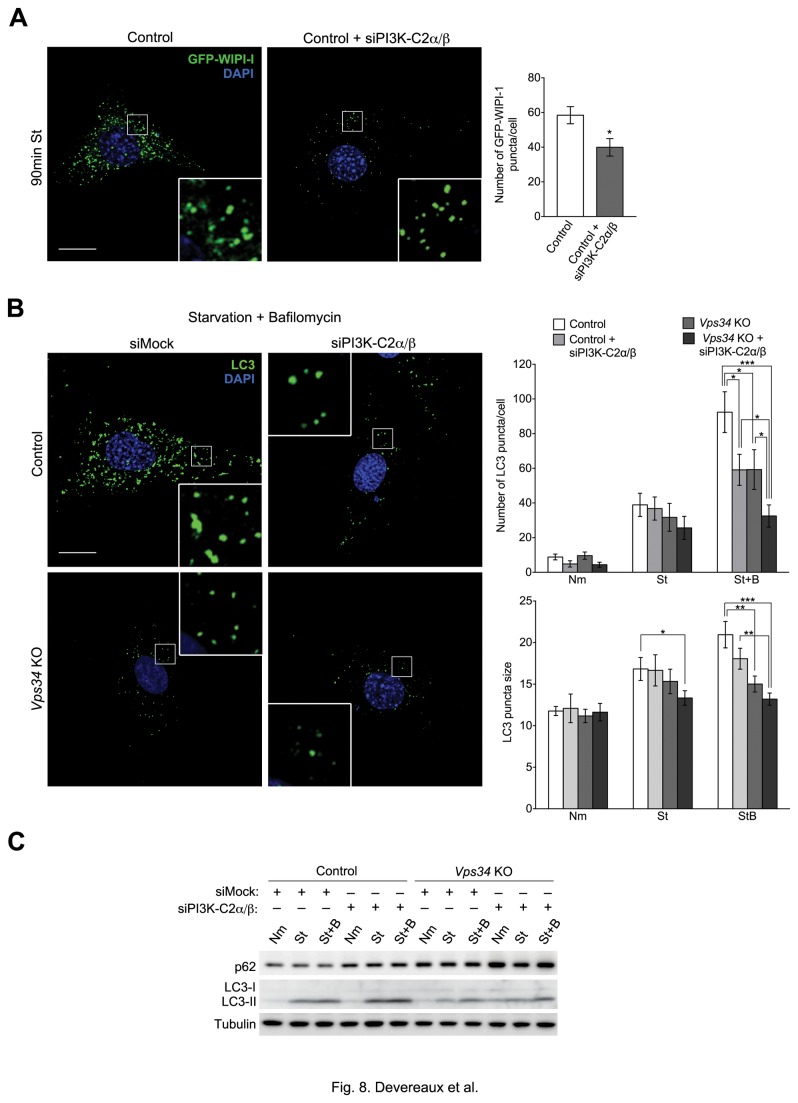
Silencing class II PI3Ks decreases autophagy in both control and *Vps34* null MEFs. (**A**) Control MEFs were transfected with mock or PI3K-C2α/β siRNA as well as GFP-WIPI-1 for 48 hrs, cultured in normal media or HBSS for 90 min and fixed. Right: Confocal microscopy images of GFP-WIPI-1 fluorescence in mock or PI3K-C2α/β siRNA-treated control cells after HBSS starvation for 90 min. Scale bar: 10 µm. Left: Quantification of the number and size (arbitrary units) of GFP-WIPI-1 puncta observed after 90 min HBSS starvation (n=11-15 cells). (**B**) Cells prepared as in (A) were fixed and immunostained. Right: Confocal microscopy images showing endogenous LC3 (green) in cells cultured in HBSS in the presence of 50 nM Bafilomycin (St+B) for 30 min. DAPI is shown in blue. Scale bar: 10 µm. Left: Quantification of the number and size (arbitrary units) of LC3 puncta observed under normal media (Nm), HBSS (St) and HBSS in the presence of Bafilomycin (St+B) conditions (n=12-19, 23-40 and 26-53 cells for Nm, St and St+B conditions, respectively). Scale bars: 10 µm.(**C**) Control and *Vps34* KO MEFs were transfected for 48 hrs with mock or PI3K-C2α/β siRNA, cultured in normal medium (N), HBSS (St) or HBSS with 50 nM Bafilomycin (St+B) for 30 min, lysed and analyzed by immunoblotting using the indicated antibodies (n=3).

Since lack of Vps34 did not abolish autophagy and PI3P production, we next examined whether class II PI3Ks could be an additional source of PI3P required to promote AP biogenesis. To specifically test for whether PI3K-C2α/β may be regulating autophagy through PI3P production, we examined GFP-WIPI-1 puncta formation upon silencing of the PI3K-C2α/β isoforms in starved control cells (Figure S4). Silencing of PI3K-C2α/β caused a ^~^30% decrease in the number of GFP-WIPI-1 puncta (Figure 8A), compared to the ^~^80% decrease observed in the Vps34-deficient cells (Figure 3), which seemingly accounts for the totality of WIPI-1 puncta in response to starvation. Thus, although the contribution of the class II PI3Ks to the formation of WIPI-1 puncta is quantitatively minor relative to that of Vps34, they appear to be significant modulators of autophagy through production of PI3P. We next examined the effect of the class II PI3Ks on LC3 localization during starvation-induced autophagy by siRNA silencing of PI3K-C2α/β isoforms in control and *Vps34* KO MEFs (Figure S4). Under normal media conditions, a trend for a decrease in LC3 puncta number was observed upon siPI3K-C2α/β-treatment of control MEFs (Figure 8B). While minor differences were observed during starvation for 30 min, changes in autophagic flux became more apparent in the presence of Baf (Figure 8*B*, S5). Indeed, knocking down class II PI3Ks in control MEFs produced a ^~^40% decrease in the number of LC3-positive puncta in response to a 30 min starvation period in the presence of Baf, a decrease of the same magnitude as that achieved by knocking out Vps34 (Figure 8B). When Vps34-deficient cells were treated with PI3K-C2α/β siRNAs, the number of LC3 puncta was further decreased relative to siPI3K-C2α/β-treated control or *Vps34* KO MEFs (Figure 8B). We also observed a decrease in LC3 puncta size in siPI3K-C2α/β-treated control MEFs that was exacerbated by the absence of Vps34 (Figure 8B). While silencing of class II PI3Ks in control and *Vps34* KO MEFs did not prevent LC3 lipidation in response to starvation, an increase in p62 was observed in *Vps34* KO MEFs treated with PI3K-C2α/β siRNA, suggesting class II PI3Ks may be playing a positive regulatory role in autophagy (Figure 8C). Importantly, these results indicate that PI3K-C2α/β positively regulate autophagy in addition to Vps34.

## Discussion

Determining the lipid signaling events required for AP formation is critical to our understanding of the regulation of autophagy. PI3P is known to play a critical role in orchestrating AP formation during starvation-induced autophagy [6,8,10-12], however, unlike lower eukaryotes, such as budding yeast, where Vps34 is the only source of PI3P, other pathways of PI3P synthesis have been described in higher eukaryotes, and mammalian cells in particular [54-56]. These include generation of PI3P by class II PI3Ks, which catalyze a PI phosphorylation like Vps34, and inositol 4-phosphatase (Inpp4) type I and II, which dephosphorylate PI(3,4)P_2_ to PI3P [57-59]. Additionally, Sac1 family members, such Figure 4 can dephosphorylate PI(3,5)P_2_ to PI3P, accounting for yet another potential source of PI3P [60,61]. Evidence that an alternative source of PI3P could potentially regulate mammalian autophagy originated from reports on conditional KO mice showing that APs can form in Vps34-deficient sensory neurons [36] and T lymphocytes [37]. Additionally, a class II PI3K isoform, *PIK3C2α*, was also identified as a ‘hit’ in a recent autophagy interaction network screen [43]. Therefore, we aimed to assess the extent of autophagy occurring in the absence of Vps34 and determine whether the class II PI3K family contributes to autophagy regulation. Here we performed a comprehensive series of autophagy assays using *Vps34* KO MEFs which confirmed that while Vps34 is important for autophagy, it is not the sole source of PI3P synthesis during autophagy. Importantly, we showed that in addition to Vps34, the class II PI3Ks contribute a pool of PI3P that positively regulates AP biogenesis.

Several conditional *Vps34* KO mouse models have been generated recently, with selective deletions in sensory neurons [36], T-lymphocytes [37-39], podocytes [41], fibroblasts, cardiomyocytes and hepatocytes [62] as well as lens cells [42]; however, the requirement for Vps34 in autophagy remains highly debated. Potential explanations for phenotype discrepancies include differential gene targeting strategies (e.g., deletion of proximal vs. distal exons), mouse genetic backgrounds, cell types and age-dependency of phenotypes. In our mouse model, we have confirmed by Western blot analysis using antibodies to both the NH_2_- and COOH-terminus of Vps34 that no residual, truncated forms of the protein remain expressed in the KO despite the fact that our strategy targets distal exons in the catalytic domain (i.e., exons 17-18) [36]. Complete loss of Vps34 is important to ensure in KO models, considering truncated forms of the protein can in some cases provide partial function or even cause dominant negative effects. We note that Jaber et al. also characterized *Vps34* KO MEFs and found a more robust defect in autophagy [62]. In addition to this apparent discrepancy perhaps due to differences in targeting strategy (e.g., proximal exon 4 targeting was targeted in Jaber et al.), it may be that differences in genetic background influence the regulation of the various PI3P synthesis pathways. Similarly, distinct cell types may differentially rely on the Vps34-dependent and -independent pathways. For instance, PI3P measurements showed that it is PI3KC2α that contributes to the majority of steady-state PI3P in endothelial cells, demonstrating such cellular differences [63]. By identifying the class II PI3Ks as an additional source of PI3P during autophagy, our study provides a potential explanation for the difference in phenotypes observed among the various Vps34 conditional KO models [63]. Lastly, we noticed that prolonged Vps34 ablation caused a progressive breakdown of the endolysosomal function. In a recent study where Vps15 (i.e., a key subunit of the Vps34 complex) was ablated in skeletal muscle, which subsequently caused a secondary loss of Vps34, similar findings were reported and reminiscent of the lysosomal storage disorders such as Pompe’s disease [51]. Importantly, like in our *Vps34* KO MEFs, AP biogenesis was still evident in *Vps15* KO skeletal muscle cells by EM; however an accumulation of APs and ALs was also observed due to secondary endolysosomal dysfunction, likely as a result of defects in AP maturation and clearance. Because endolysosomal dysfunction impacts the functionality of autophagy, it can potentially confound the analysis and interpretation of the early autophagic role of Vps34. By performing our analysis upon acute Vps34 ablation prior to a collapse of the endolysosomal system, we aimed to more accurately delineate the specific requirements for Vps34 in the autophagy process.

Synthesis of PI3P by both class II and III PI3Ks during the same physiological process may be an emerging theme. In fact, a recent study in *C. elegans* reported the class II ortholog, PIK-1, produces an initial pool of PI3P on nascent phagosomes prior that of Vps34, which is responsible for the sustained production of PI3P on these organelles [64]. While the *C. elegans* data indicates that PIK-1 and Vps34 act sequentially, we propose that in the mammalian system the effects of Vps34 and class II PI3Ks are additive, demonstrated by p62 accumulation, LC3 puncta formation and WIPI-1 recruitment, suggesting they act in parallel pathways. The class II PI3Ks are an understudied class of PI3Ks and currently there is growing interest in determining whether they cooperate or function redundantly with Vps34. The α isoform has been implicated in processes such as glucose transport, insulin secretion and endocytosis, whereas the β isoform has been shown to promote cell migration, cell-cycle progression, growth and survival [54,63,65]. Less is known about the PI3KC2γ isoform, whose expression may be limited to the liver [54]. While class II isoforms can synthesize PI3P, PI(3,4)P_2_ and PI(3,4,5)P_3_ *in vitro*, PI appears to be the preferential substrate even for the α isoform which is able to increase its activity toward PI4P and PI(4,5)P_2_ substrates in the presence of clathrin [54,66]. The majority of studies in intact cells have reported PI3P synthesis by the class II PI3Ks at the plasma membrane, in the nucleus, the Golgi complex, phagosomes and endosomes, although a recent study revealed a role for the α isoform in PI(3,4)P_2_ synthesis at the plasma membrane to promote clathrin-mediated endocytosis [54,65]. Here we provide novel evidence that class II PI3K α and β isoforms supply a pool of PI3P during AP biogenesis, based on the partial loss of WIPI-1 puncta upon their silencing. Because WIPI-like proteins, such as Atg18, do not bind efficiently to PI(3,4)P_2_ [67], this lipid is not the likely product of class II PI3Ks in the context of autophagy, although participation of PI(3,4)P_2_ in the autophagy process, including AP biogenesis, cannot be ruled out. Although the pool of PI3P produced by the class II PI3Ks during autophagy is smaller than that produced by Vps34, knocking down PI3K-C2α/β reduces LC3 puncta formation to the same extent as knocking out Vps34, indicating class II PI3Ks still plays a significant role in starvation-induced autophagy. Importantly, although the class II PI3Kα isoform was identified as a "hit" in a genome wide screen looking for new components of the autophagy network and a potential interactor of Atg7 and MAP1LC3A in U2OS cells [43], the same isoform was not identified as a hit in an independent study aiming to identify new regulators of autophagy among genes harboring FYVE and PX domains in 293T cells [68]. In our study, silencing of both class II PI3K α and β isoforms was required to cause an autophagy phenotype, suggesting there is some level of redundancy within the class II PI3K family.

Our studies indicate that together Vps34 and the class II contribute to the majority of PI3P synthesis during autophagy. Notably, we observed few LC3 puncta in *Vps34* KO MEFs upon concomitant knockdown of the class II PI3Ks, suggesting a small amount of autophagy may still be occurring in the absence of Vps34 and the class II PI3Ks. Whether PI3P can be produced via a third pathway, such as dephosphorylation of PI(3,4,5)P_3_ by PI 5-phosphatases and PI 4-phosphatases (Inpp4 I and II) will require further investigation [59]. As mentioned above, contribution of these different PI3P synthesis pathways to autophagy may be largely determined by cell type and genetic background. It will be important to determine whether the physiological context of autophagy stimulation, site of AP biogenesis and type of cargo dictate which pathways of PI3P synthesis are used.

As previously shown in other studies, including in yeast [27,28], our results indicate that Vps34 is not essential for LC3 lipidation *per se*. In fact, the *Vps34* conditional KO models have reported findings that range from decreased LC3-II levels [38]; see also Figure 4A), no change [37] or even an increase in LC3-II levels [36,39-42]. The findings are consistent with the view that Vps34 and more generally, PI3P, are involved in spatially restricting where LC3 lipidation (and AP biogenesis) occur, rather than controlling the enzymatic conjugation reaction itself. Importantly, our work suggests that pharmacological experiments utilizing wortmannin or 3MA should be interpreted with caution, as the robust effects these drugs typically exert on autophagy are likely to be caused in part by targets distinct from Vps34. We found that LC3-II levels were drastically reduced during starvation in both control and *Vps34* KO MEFs treated with wortmannin and 3MA. Although class I PI3K are known to be susceptible to these inhibitors and may effect the production of PI(3,4,5)P_3_ and its subsequent dephosphorylation by 4- and 5-phophatases to PI3P, the alternate targets of wortmannin are unlikely to be class II PI3Ks, as these enzymes (particularly the α isoform) are less sensitive to wortmannin [69] and silencing of class II PI3Ks does not alter LC3-II levels (Figure 8C). Future studies identifying the additional targets of wortmannin and 3MA will be helpful to our understanding the regulation of LC3 conjugation during autophagy as well as to potentially discover new targets for modulating autophagy.

Overall our study highlights the complexity and plasticity of PI3P metabolism in mammalian cells and provides strong impetus for future studies exploring the precise role of Vps34-independent sources of PI3P in autophagy, including class II PI3Ks and potentially, the Inpp4 pathway, and their regulation during this essential process. Lastly, reports of noncanonical autophagy in the field have suggested that autophagy can occur independently of known factors, such as Beclin 1 [70]. The extent to which autophagy can occur without PI3P synthesis altogether remains undetermined.

## Materials and Methods

### Ethics Statement

Animals were used in full compliance with National Institutes of Health/ Institutional Animal Care and Use Committee guidelines. Specifically, adult mice were sacrificed via inhalation of carbon dioxide, followed by cervical dislocation, while embryos derived from the euthanized mothers were decapitated after inducing hypothermia on ice. The animal protocols were approved by the Committee on the Ethics of Animal Experiments of Columbia, Yale and Duke Universities.

### Generation of *Vps34* KO MEFs

Primary mouse embryonic fibroblasts (MEFs) were generated from embryonic day 13.5 *Vps34* (*pik3c3*) *Flox/Flox* mouse embryos in which exons 17/18, encoding the catalytic domain, were flanked by *loxP* sites, as described previously [36]. MEFs were immortalized by multiple passaging and were used for experiments after 25 passages. Control Vps34^Flox/Flox^ (Control) and *Vps34* ^*-/-*^ (KO) cells were generated by infecting cells with a lentivirus carrying either a catalytically active Cre recombinase or catalytically-dead Cre recombinase (control), respectively. Both Cre recombinases encoded two nuclear localization sequences (NLS) and were fused to either eGFP or Tomato moieties containing a third NLS for enhanced nuclear targeting. Lentivirus was produced in human embryonic kidney 293T cells according to Ho et al. with slight modifications [71]. Virus-containing media was harvested 72 hrs after transfection, centrifuged at 2,800xg for 5 min, filtered through a 0.45mm filter (Millipore) and supplemented with 10µg/ml polybrene (Sigma-Aldrich) prior to adding to MEFs. Infection rate was estimated by determining the percentage of cells with nuclear EGFP or Tomato-fluorescent positivity. In addition, *Vps34* ^*Flox/Flox*^; Cre-ER MEFs were generated for experiments requiring larger amounts of cells such as HPLC biochemistry studies using the protocol mentioned above. MEFs contain a Cre-estrogen receptor (Cre-ER) fusion protein whereby Cre activity is inducible by tamoxifen (4-HT [72]. To generate *Vps34* KO and control cells, *Vps34* ^*Flox/Flox*^; Cre-ER MEFs were incubated with either 3µM 4-hydroxytamoxifen (4-HT) (Sigma-Aldrich) diluted in ethanol or vector alone for 72hrs followed by culturing in normal growth media. In both MEF cell lines, KO efficiency was monitored by Western blot analysis using antibodies to the NH_2_- and COOH-termini of Vps34. A minimum of 10 days post-infection or tamoxifen treatment was required to abolish the expression of Vps34, therefore, all experiments were performed upon acute gene ablation 10 days after infection, unless indicated otherwise.

### Cell culture and transfection

MEFs were cultured in DMEM-Glutamax (Invitrogen) supplemented with 10% fetal bovine serum (FBS), penicillin (200 units/mL)/streptomycin (200 µg/mL) (Invitrogen) at 37 °C under 5% CO2. For starvation assays, MEFs were cultured in Hanks’ buffer (Invitrogen) for the indicated times. MEFs were transiently transfected using the Lipofectamine RNAiMAX (Invitrogen) and/or AMAXA MEF I Nucleofector Kit (Lonza) according to the manufacturer’s instructions. Cells were incubated with siRNA for 48 hrs and efficient silencing was confirmed by Western blot for each experiment. Plasmids were transiently expressed for 24 hrs when transfected alone or 48 hrs when co-transfected with siRNA.

### Reagents and antibodies

The following compounds were used in autophagy assays: bafilomycin A1 (50nm, Wako), 3-methyladenine (10mM, Sigma), wortmannin (100nM, Billerica). Primary antibodies used for Western blotting include: anti-pan-actin mouse polyclonal antibody (1:4000 dil, Novus), anti-Atg5 rabbit polyclonal antibody (1:500 dil, Abgent; to detect free form), anti-Atg5 rabbit polyclonal antibody (1:1,000 dil, Sigma; to detect conjugated form), anti-Atg14 rabbit polyclonal antibody (1:500 dil, generous gift from Dr. Zhenyu Yue), anti-Beclin mouse polyclonal antibody (1:1000 dil, BD Biosciences), anti-EEA1 mouse polyclonal antibody (1:500 dil, Thermoscientific), anti-EGFR rabbit polyclonal antibody (1:1000 dil, Millipore), anti-LC3 rabbit polyclonal antibody (1:1000 dil, Novus), anti-p62/SQSTM1 mouse monoclonal antibody (2C11; 1:2000 dil, Abnova), anti-PI3KC2α rabbit polyclonal antibody (1:1000 dil; Santa Cruz), anti-PI3KC2β mouse polyclonal antibody (1:1000 dil, BD Biosciences), anti-Rab5 621.3 mouse monoclonal antibody (1:500 dil, Synaptic Systems), anti-Rubicon rabbit polyclonal antibody (1:500 dil, generous gift from Dr. Zhenyu Yue), anti-β-tubulin mouse polyclonal antibody (1:4000 dil, Sigma), anti-UVRAG mouse monoclonal antibody (1H4, 1:500 dil, MBL), anti-Vps15/p150 mouse monoclonal antibody (M02, 1:1000 dil, Abnova), anti-Vps26 rabbit polyclonal antibody (1:1000 dil, Abcam), anti-Vps34/PI3KC3 rabbit monoclonal antibody (D4E4, 1:1,000 dil, Cell Signaling Technology; to amino terminal sequence), anti-Vps34/PI3KC3 rabbit monoclonal antibody (D9A5; 1:1,000 dil; Cell Signaling Technology; to C-terminal sequence) and anti-Vps35 mouse polyclonal antibody (1:1000 dil, Abcam). Secondary antibodies used for western blot include HRP conjugated anti-guinea pig (1:3000 dil, Santa Cruz), anti-mouse (1:3000 dil, Biorad), and anti-rabbit (1:3000 dil, Biorad). Immunofluorescence experiments were conducted using the following primary antibodies: anti-Atg16L1 rabbit polyclonal antibody (1:200 dil, Cosmo), anti-EEA1 goat polyclonal antibody (1:200 dil, Santa Cruz), anti-Giantin rabbit polyclonal antibody (1:1000 dil, Covance), anti-LAMP1 rabbit polyclonal antibody (1:500 dil, Abcam), anti-LC3 mouse monoclonal antibody (4E12, 1:100 dil, MBL), anti-myc mouse monoclonal antibody (9E10, 1:400 dil, Roche), anti-p62/SQSTM1 guinea pig polyclonal antibody (1:1000 dil, Progen) and anti-PDI mouse monoclonal antibody (1D3; 1:50 dil, Stressgen). Fluorescent secondary antibodies used include: Alexa Fluor 647 donkey anti-goat (1:100 dil, Jackson Immunoresearch), Alexa Fluor 647 donkey anti-guinea pig (1:100 dil, Jackson Immunoresearch), Alexa Fluor 488 donkey anti-mouse (1:200 dil, Molecular Probes), Alexa Fluor 555 donkey anti-mouse (1:200 dil, Molecular Probes), Alexa Fluor 647 donkey anti-mouse (1:100 dil, Jackson Immunoresearch), Alexa Fluor 488 donkey anti-rabbit (1:200 dil, Molecular Probes), Alexa Fluor 555 donkey anti-rabbit (1:200 dil, Molecular Probes) and Alexa Fluor 647 donkey anti-rabbit (1:100 dil, Jackson Immunoresearch).

### Plasmids and RNAi

RFP-2x-FYVE^HRS^ and GFP-2x-FYVE^HRS^ plasmids were gifts from Harald Stenmark (University of Oslo, Norway). To generate GFP-4x-FYVE^HRS^, the FYVE domain of HRS was amplified with XhoI/SalI sites and cloned twice into the XhoI site of the GFP-2x-FYVE^HRS^ plasmid. GFP-WIPI-1 was a gift from Sharon Tooze (Cancer Research UK). Second generation lentiviral packaging plasmids VSVg and ∆8.9 were gifts from Peter Scheiffele (University of Basel), whereas the EGFP-tagged active, full-length Cre recombinase and catalytically inactive, truncated Cre FUGW lentiviral constructs were a gift from Thomas Sudhof (Stanford University, California) [73]. Active and inactive Tomato-tagged Cre FUGW lentiviral constructs were generated by EcoR1/BsrG1 replacement of EGFP in the CRE FUGW vector with a PCR-amplified Tomato sequence that included EcoR1/BsrG1 overhangs and retained the third N-terminal NLS by using the following primers: forward, 5’-AAATAAGAATTCACAACCATGGTGAAGCGACCAGCAGCAACAAAGAAGGCA GGACAAGCAAAGAAGAAGAAGCTCGTGAGCAAGGGCGAGGAGG-3’ and reverse, 5’-AAATAATGTACAGCTCGTCCATGCC-3’. Gene silencing was achieved using pre-designed siRNA sequences against murine *pik3c2α* (5’-TTGGCAGAAATTATAAACTTA-3’; SI02671011) and murine *pik3c2β* (5’-CTGGCTCTGATCCCACCCTAA-3’; SI00935928) (Qiagen). As a negative control, an siRNA scramble sequence (5’-AATTCTCCGAACGTGTCACGT-3’; 1027310) was used.

### SDS-PAGE and Western blotting

MEFs cultured in 60 or 100mm plates were lysed in lysis buffer (50 mM Tris–HCl (pH 7.2), 250 mM NaCl, 0.1% NP-40, 2 mM EDTA, 10% glycerol) containing protease (Complete Mini, EDTA-Free, Protease Inhibitor Cocktail; Roche) and phosphatase (phosSTOP; Roche) inhibitors. Extracts were centrifuged at 16,000x*g* for 30 min at 4 °C and the protein concentration was measured using the Coomassie Plus Protein Assay Kit (Thermo-Scientific). Proteins were separated by SDS–PAGE and transferred to PVDF (Immobilon-P; Millipore) or nitrocellulose membranes (iBlot Transfer Stack; Invitrogen). Membranes were probed with the indicated primary antibodies and the appropriate HRP-conjugated secondary antibodies. Immunoreactive protein bands were visualized and imaged using a chemiluminescent HRP substrate kit (Immobilon Western; Millipore) and the Fuji LAS4000 Imaging Unit (GE Healthcare), respectively. Densitometric quantification was performed using the ImageJ software (NIH).

### EGFR degradation assay

MEFs were grown in 60mm dishes and serum-starved overnight in DMEM-Glutamax (Invitrogen). Cells were then stimulated with 100 ng/mL EGF (Millipore) in serum-free medium and harvested at the indicated time points.

### Immunofluorescence microscopy

MEFs grown on coverslips were fixed with 4% paraformaldehyde for 20 min at room temperature. After permeabilization with 200 µg/ml digitonin (Invitrogen) in PBS for 10 min, cells were incubated with the specified primary antibodies for 1 hr at room temperature. Subsequently, cells were incubated with the appropriate Alexa-Fluor or Cy5-conjugated secondary antibodies for 1 hr at room temperature and coverslips were mounted in DAPI Fluoromount G (Southern Biotech). Images were acquired by confocal laser scanning microscopy (Zeiss LSM-700) and analyzed with Zeiss Zen and ImageJ Software (NIH). The number of LC3-positive compartments and their surface areas (expressed as number of pixels per field) were normalized to the number of cells in each field. The average size was obtained by dividing the surface area of the LC3-positive compartment (in pixel^2^) by the number of LC3 puncta. Similar measurements were made for Atg16L1- and p62-positive compartments. The colocalization between p62 and LC3 was calculated as the number of pixels overlapping in the two channels per cell.

### Conventional electron microscopy and morphometric analysis

MEFs cultured on plastic coverslips (LF1, Sumitomo Bakelite, Tokyo, Japan) were fixed in 2.5% glutaraldehyde (Electron Microscopy Sciences) in 0.1 M sodium phosphate buffer (PB) (pH 7.4) for 2 hrs at room temperature. After washing with PB, the cells were post-fixed in 1% OsO4 in PB for 60 min at room temperature and washed with distilled water before being dehydrated in a series of graded ethanol solutions and embedded in epoxy resin. Ultra-thin sections were doubly stained with uranyl acetate and lead citrate and observed under an H7600 electron microscope (Hitachi, Tokyo, Japan). Electron micrographs were taken of thin sections from three different parts of the plastic cover slips using a H7600 electron microscope (Hitachi, Tokyo, Japan) equipped with ORIUS™ SC200W 2 k x 2 k TEM CCD camera (Gatan Inc. CA) at a magnification of 8,000. Thirty electron micrographs were taken per condition and analyzed by morphometry. The number and surface area of autophagosomes/amphisomes (AP) and autolysosomes/ lysosomes (AL) were measured using the software MacSCOPE 2.5 (Mitani Corporation, Fukui, Japan). The surface area of APs and ALs was normalized to the cytoplasmic area.

### Immunoelectron microscopy

The pre-embedding gold enhancement immunogold method was used for immunoelectron microscopy as described previously [74] with slight modifications. Briefly, MEFs cells were cultured on plastic coverslips (LF1, Sumitomo Bakelite, Tokyo, Japan) and fixed in 4% paraformaldehyde in PB for 2 hrs at room temperature. After permeabilizing in 0.25% saponin diluted in PB for 30 min and blocking in 0.1% saponin, 10% bovine serum albumin, 10% normal goat serum and 0.1% cold water fish skin gelatin diluted in PB for 30 min, cells were incubated overnight in mouse monoclonal anti-LC3 antibody [75,76] (clone LC3 1703, Cosmo Bio. Co. LTD Tokoyo, Japan) diluted in blocking solution. Subsequently, MEFs were incubated with colloidal gold (1.4 nm in diameter, Nanoprobes, New York, NY)-conjugated anti-mouse IgG Fab fragments diluted in the blocking solution for 2 hrs. The signal was intensified using a gold enhancement kit (GoldEnhance EM, Nanoprobes) for 2 min at room temperature. Cells were post-fixed in 1% OsO4 containing 1.5% potassium ferrocyanide at room temperature and processed as described above for conventional EM.

### Intracellular protein turnover

The long-lived protein degradation assay was adapted from previously reported protocols [77,78]. Control and *Vps34* KO MEFs were plated in 6-well plates. The following day, MEFs were labeled with [^14^C]Valine (0.1µCi/ml, PerkinElmer) for 18 hrs at 37 °C. Cells were washed three times with PBS before chasing with radioactivity-free medium containing an excess of unlabeled valine (10mM, Sigma) for 3 hrs to allow for short-lived protein degradation. Again, cells were washed three times with PBS and cultured in complete medium or in serum-free medium (HBSS) either alone or in the presence of 3-methlyadenine (10mM, Sigma) or NH_4_Cl (15mM, Sigma). A fraction of the medium was taken at different time points and precipitated with trichloroacetic acid (final concentration 15% v/v, Sigma) in order to separate [^14^C]Valine incorporated into secreted proteins from that of free [^14^C]Valine released after intracellular proteolysis. After 4 hrs, cells were washed three times in PBS, lysed (0.1M NaOH, 0.1% Na deoxycholate) and TCA-precipitated. TCA-soluble and total cell radioactivity were measured by liquid scintillation counting. Proteolysis was estimated as the ratio of TCA-soluble radioactivity to the total cell radioactivity. Specifically, total lysosomal degradation was calculated as a percentage of protein degradation sensitive to NH_4_Cl. Contribution of macroautophagy calculated as a percentage of lysosomal protein degradation sensitive to 3MA. Microautophagy and/or chaperone-mediated autophagy (CMA) account(s) for the difference between total lysosomal degradation and macroautophagy-associated protein degradation.

### Quantification of phosphoinositide levels by HPLC analysis

Control and *Vps34* KO cells produced via the Cre-ER system were incubated with inositol-free DMEM (MP Biomedicals) supplemented with 10% dialyzed FBS (Invitrogen), L-glutamine, glucose and 5 µCi/ml ^3^H-myo-inositol (MP Biomedicals) 24 hrs before transfection with siRNA duplexes. After transfection, cells were again incubated with 5 µCi/ml ^3^H-myo-inositol (MP Biomedicals) in inositol-free DMEM (MP Biomedicals) for an additional 48hrs. Prior to lipid extraction, cells were washed twice with PBS and incubated 15 minutes in inositol-free DMEM (MP Biomedicals) supplemented with 10% dialyzed FBS, glutamine and glucose. Cells were treated with 0.7 ml 4.5% perchloric acid in ice for 15 mins, scraped and centrifuged. Pellets were washed twice with ice-cold 1 ml 0.1 M EDTA, and deacylated as described [79]. Deacylated phosphoinositides were then separated using high performance liquid chromatography (Shimadzu Scientific Instruments). Peaks were identified using deacylated 32P-standards and detected by an online flow scintillation analyzer (B-RAM, IN/US) [80].

### Statistical analysis

Quantitative results represent three independent experiments (unless indicated otherwise) and values denote mean±s.e.m., as indicated in each figure. Statistical analysis was performed using a two-tailed, equal variance Student’s *t*-test, except for data shown in Figure 1C, where the one-sample *t*-test was used. *P*-values of < 0.05 (*), < 0.01 (**), < 0.001 (***) were determined to be statistically significant. All statistical data were calculated with the GraphPad Prism software.

## Supporting Information

Figure S1 **Acute and chronic loss of Vps34 differentially affects the endo-lysosomal system.** (**A**) Western blot using antibodies directed to the COOH-terminus of Vps34 in control and *Vps34* KO cell extracts. (**B**) Confocal analysis of control and *Vps34* KO MEFs immunostained for the following organelle markers: PDI, Giantin, EEA1 and LAMP1 (red). Nuclear inactive and active GFP-CRE is shown in green. Scale bar: 10 µm. (**C**) Right: DIC image of control and *Vps34* KO cells on day 14 post-infection. Left: Confocal analysis of LAMP1, a late endosomal/lysosomal marker, immunostaining (red) in control and *Vps34* KO MEFs on day 14 post-infection. DAPI is shown in blue. Scale bar: 10 µm. (**D**) Western blot analysis of Epidermal Growth Factor Receptor (EGFR) degradation in control and *Vps34* KO MEFs on day 10 and day 14 post-infection. MEFs were serum starved overnight and stimulated with EGF (100ng/ml) for the indicated times. EGFR protein level is quantified relative to tubulin and represented as a percent EGFR at time 0 (n=4 and 3 for day 10 and 14, resp.).
(TIF)

Figure S2 **Prolonged ablation of Vps34 causes an increase in p62 levels.** (**A**) Right: Western blot analysis of p62 levels in control and *Vps34* KO cells 10 to 13 days post-infection with either inactive (Δ) or active (CRE) CRE-lentiviruses, respectively. Left: Quantification of protein signal intensities showing relative p62 levels normalized to actin (n=5). (**B**) Immunofluorescence showing endogenous p62 (green) in control and *Vps34* KO MEFs on day 14 post-infection. Nuclear inactive and active tomato-CRE are shown in red. Scale bar: 10 µm.
(TIF)

Figure S3 **PI3K inhibitors 3-methyladenine and wortmannin block LC3-lipidation independently of Vps34.** (**A**) Western blot analysis showing LC3-II levels in WT and *Vps34* KO MEFs upon 90 min of HBSS starvation alone or in the presence of wortmannin (Wort, 100 nM) or 3-methyladenine (3MA, 10 mM) and with or without Bafilomycin (Baf or simply B, 50 nM). (**B**) Quantification of the percent inhibition of LC3 conversion by each PI3K inhibitor during starvation in control and *Vps34* KO MEFs compared to starvation alone for Bafilomycin-treated conditions (n=3).
(TIF)

Figure S4 **Silencing class II PI3Ks in control and *Vps34* KO MEFs.** Western blot analysis demonstrating protein levels in control and *Vps34* KO MEFs transfected for 48 hrs with mock or PI3K-C2α/β siRNA. Efficiency of Vps34 ablation obtained by 4-HT or CRE lentivirus treatment was consistent. Silencing of PI3K-C2α/β in either KO model was achieved with comparable efficiency.
(TIF)

Figure S5 **LC3 puncta formation during starvation-induced autophagy upon silencing class II PI3K in control and *Vps34* KO MEFs.** Control and *Vps34* KO MEFs were transfected for 48 hrs with mock or PI3K-C2α/β siRNA, cultured in normal medium (N) or HBSS (St) for 30 min, fixed and immunostained. Representative confocal microscopy images showing endogenous LC3 (green) in cells cultured in normal media (top) or HBSS starvation conditions for 30min (bottom). DAPI is shown in blue. Scale bar: 10µm.
(TIF)
