# Supplementary figures and images for: Regulation of Mammalian Autophagy by Class II and III PI 3-Kinases through PI3P Synthesis
Source: PLoS One. 2013 Oct 3;8(10):e76405. doi: 10.1371/journal.pone.0076405 (PMC3789715; doi:10.1371/journal.pone.0076405)

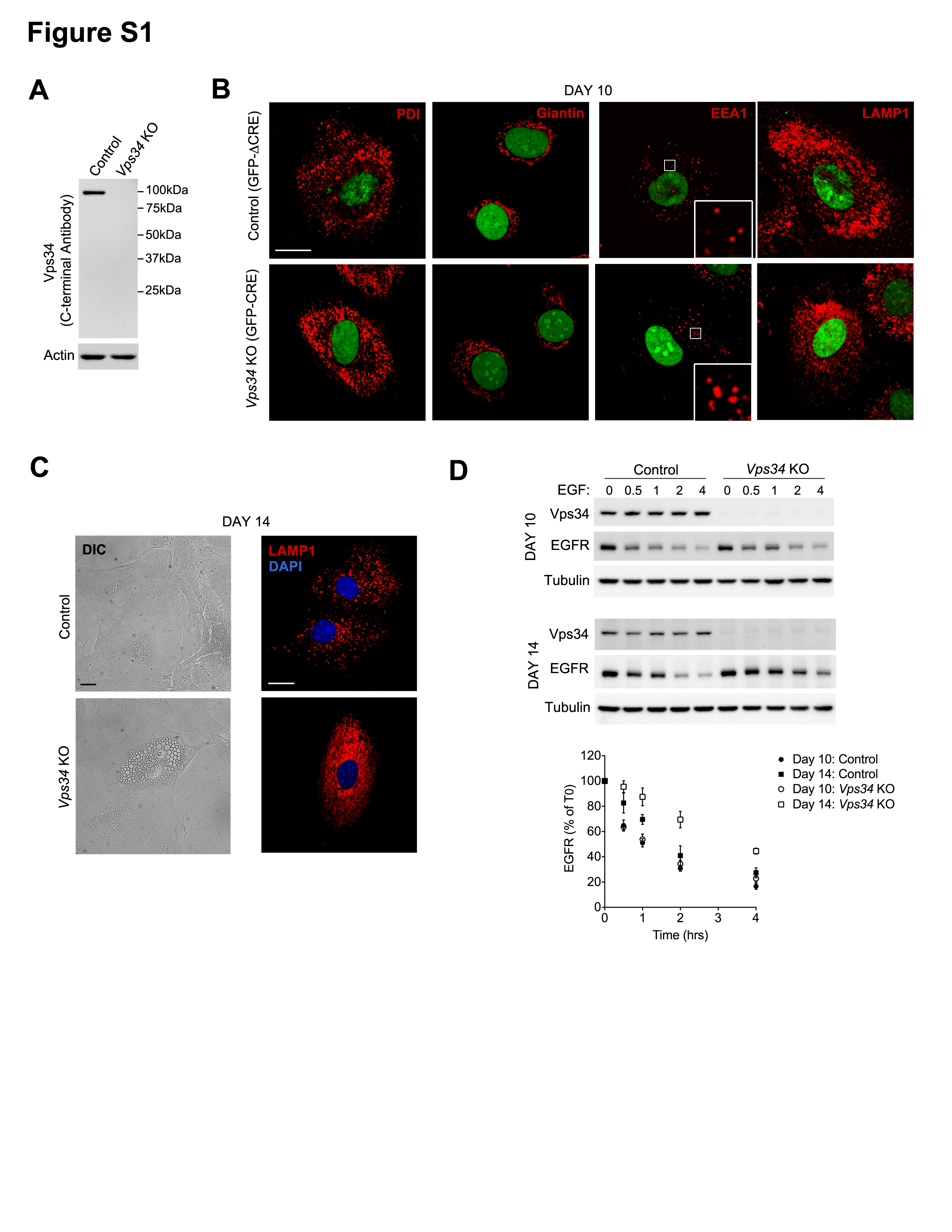

Supplement: Figure S1 — Acute and chronic loss of Vps34 differentially affects the endo-lysosomal system. (A) Western blot using antibodies directed to the COOH-terminus of Vps34 in control and Vps34 KO cell extracts. (B) Confocal analysis of control and Vps34 KO MEFs immunostained for the following organelle markers: PDI, Giantin, EEA1 and LAMP1 (red). Nuclear inactive and active GFP-CRE is shown in green. Scale bar: 10 µm. (C) Right: DIC image of control and Vps34 KO cells on day 14 post-infection. Left: Confocal analysis of LAMP1, a late endosomal/lysosomal marker, immunostaining (red) in control and Vps34 KO MEFs on day 14 post-infection. DAPI is shown in blue. Scale bar: 10 µm. (D) Western blot analysis of Epidermal Growth Factor Receptor (EGFR) degradation in control and Vps34 KO MEFs on day 10 and day 14 post-infection. MEFs were serum starved overnight and stimulated with EGF (100ng/ml) for the indicated times. EGFR protein level is quantified relative to tubulin and represented as a percent EGFR at time 0 (n=4 and 3 for day 10 and 14, resp.). (TIF) [file pone.0076405.s001.tif]

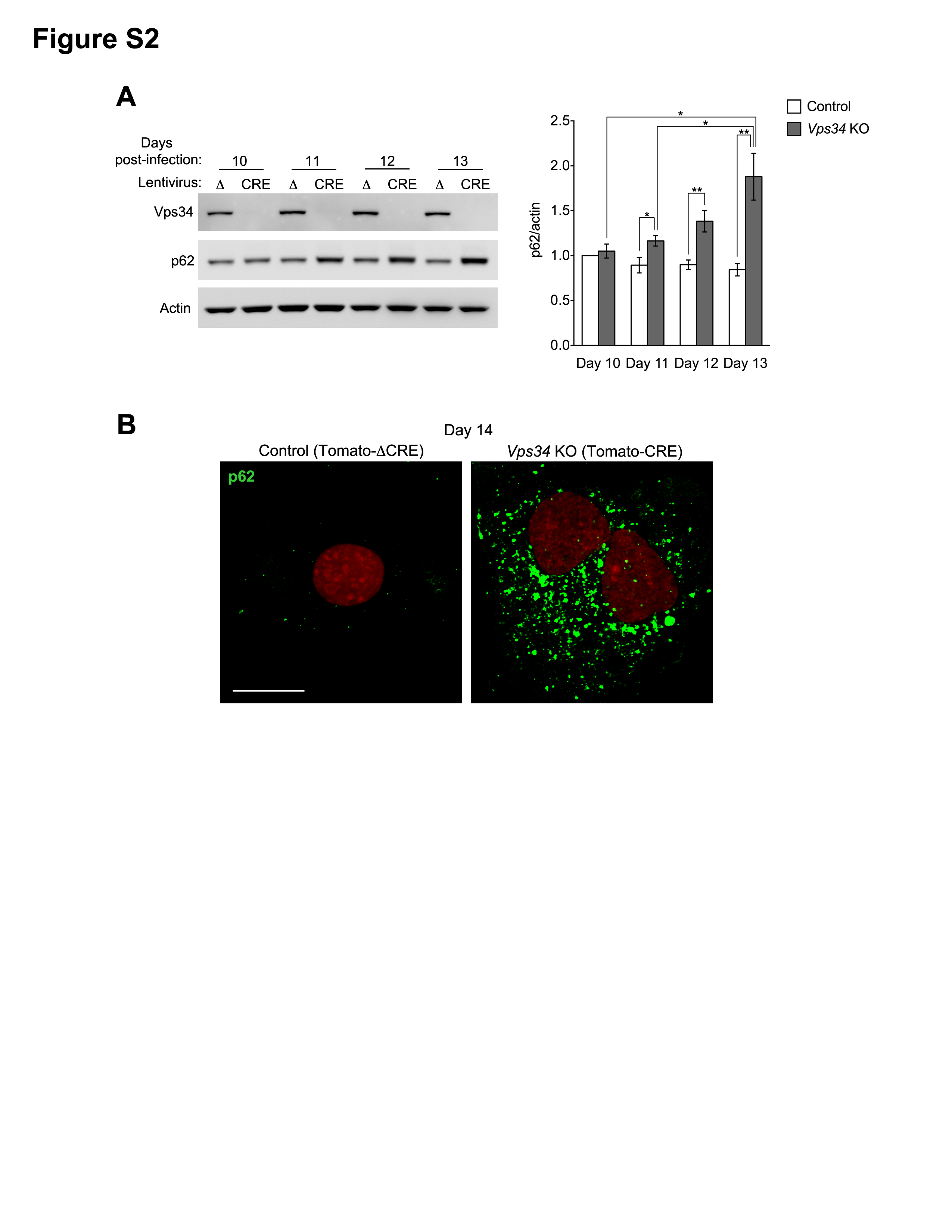

Supplement: Figure S2 — Prolonged ablation of Vps34 causes an increase in p62 levels. (A) Right: Western blot analysis of p62 levels in control and Vps34 KO cells 10 to 13 days post-infection with either inactive (Δ) or active (CRE) CRE-lentiviruses, respectively. Left: Quantification of protein signal intensities showing relative p62 levels normalized to actin (n=5). (B) Immunofluorescence showing endogenous p62 (green) in control and Vps34 KO MEFs on day 14 post-infection. Nuclear inactive and active tomato-CRE are shown in red. Scale bar: 10 µm. (TIF) [file pone.0076405.s002.tif]

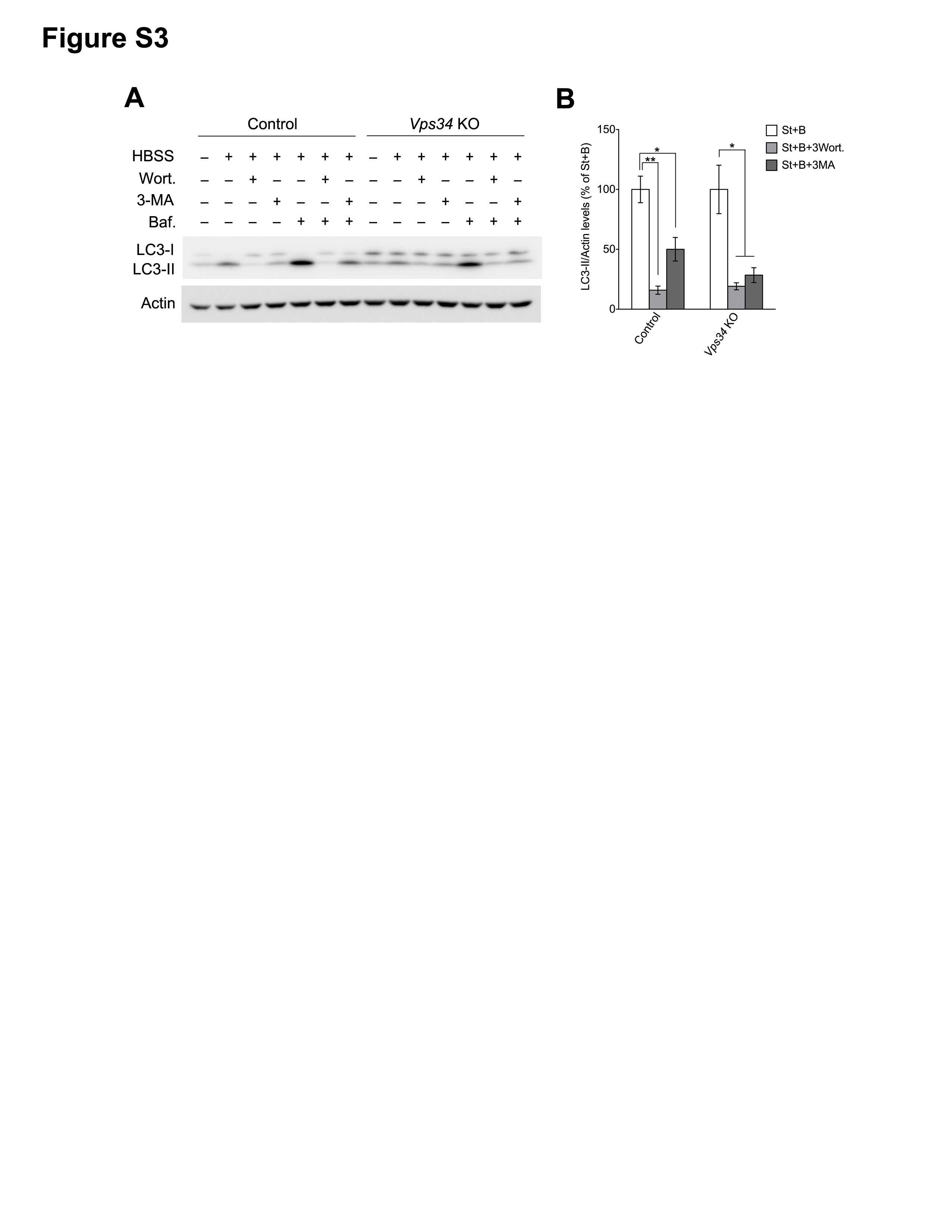

Supplement: Figure S3 — PI3K inhibitors 3-methyladenine and wortmannin block LC3-lipidation independently of Vps34. (A) Western blot analysis showing LC3-II levels in WT and Vps34 KO MEFs upon 90 min of HBSS starvation alone or in the presence of wortmannin (Wort, 100 nM) or 3-methyladenine (3MA, 10 mM) and with or without Bafilomycin (Baf or simply B, 50 nM). (B) Quantification of the percent inhibition of LC3 conversion by each PI3K inhibitor during starvation in control and Vps34 KO MEFs compared to starvation alone for Bafilomycin-treated conditions (n=3). (TIF) [file pone.0076405.s003.tif]

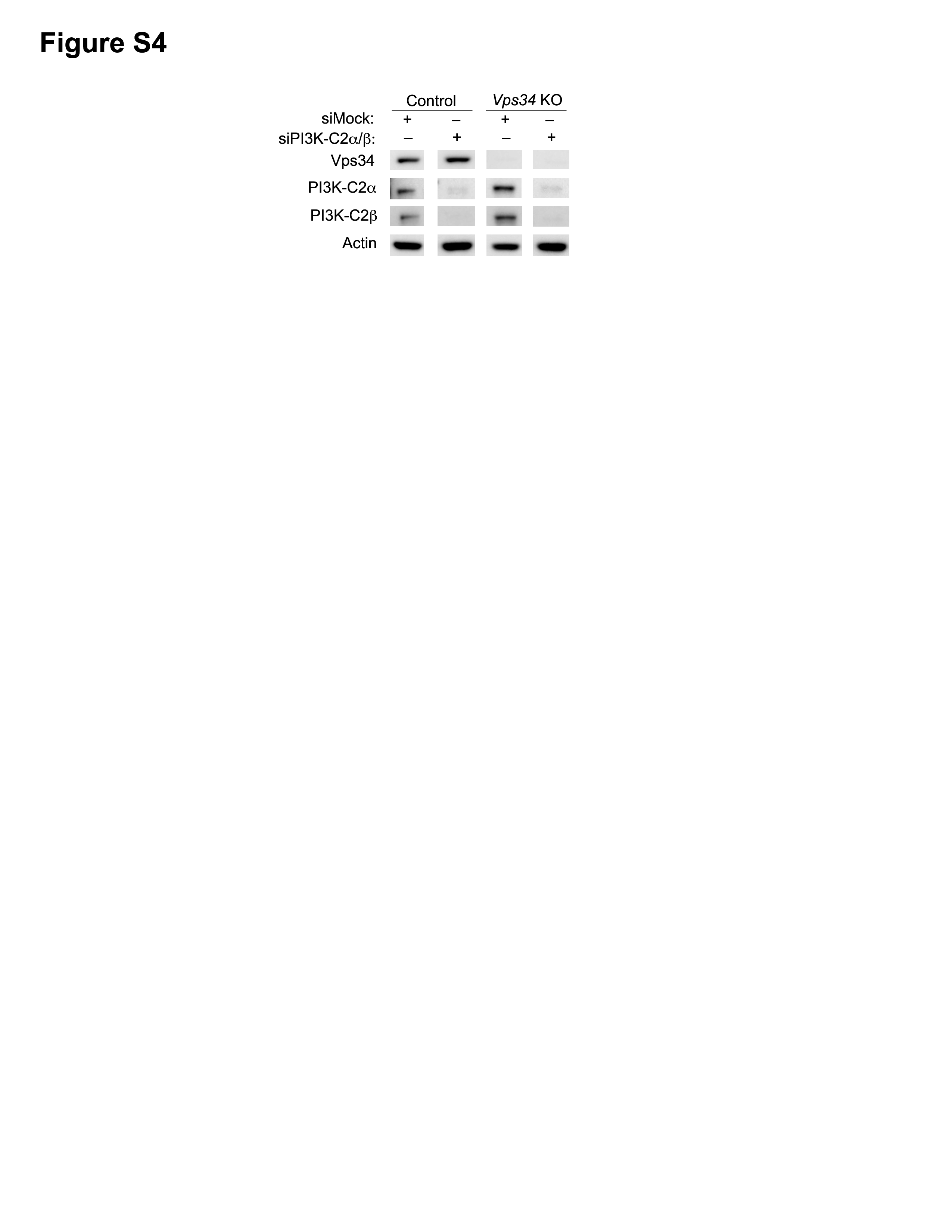

Supplement: Figure S4 — Silencing class II PI3Ks in control and Vps34 KO MEFs. Western blot analysis demonstrating protein levels in control and Vps34 KO MEFs transfected for 48 hrs with mock or PI3K-C2α/β siRNA. Efficiency of Vps34 ablation obtained by 4-HT or CRE lentivirus treatment was consistent. Silencing of PI3K-C2α/β in either KO model was achieved with comparable efficiency. (TIF) [file pone.0076405.s004.tif]

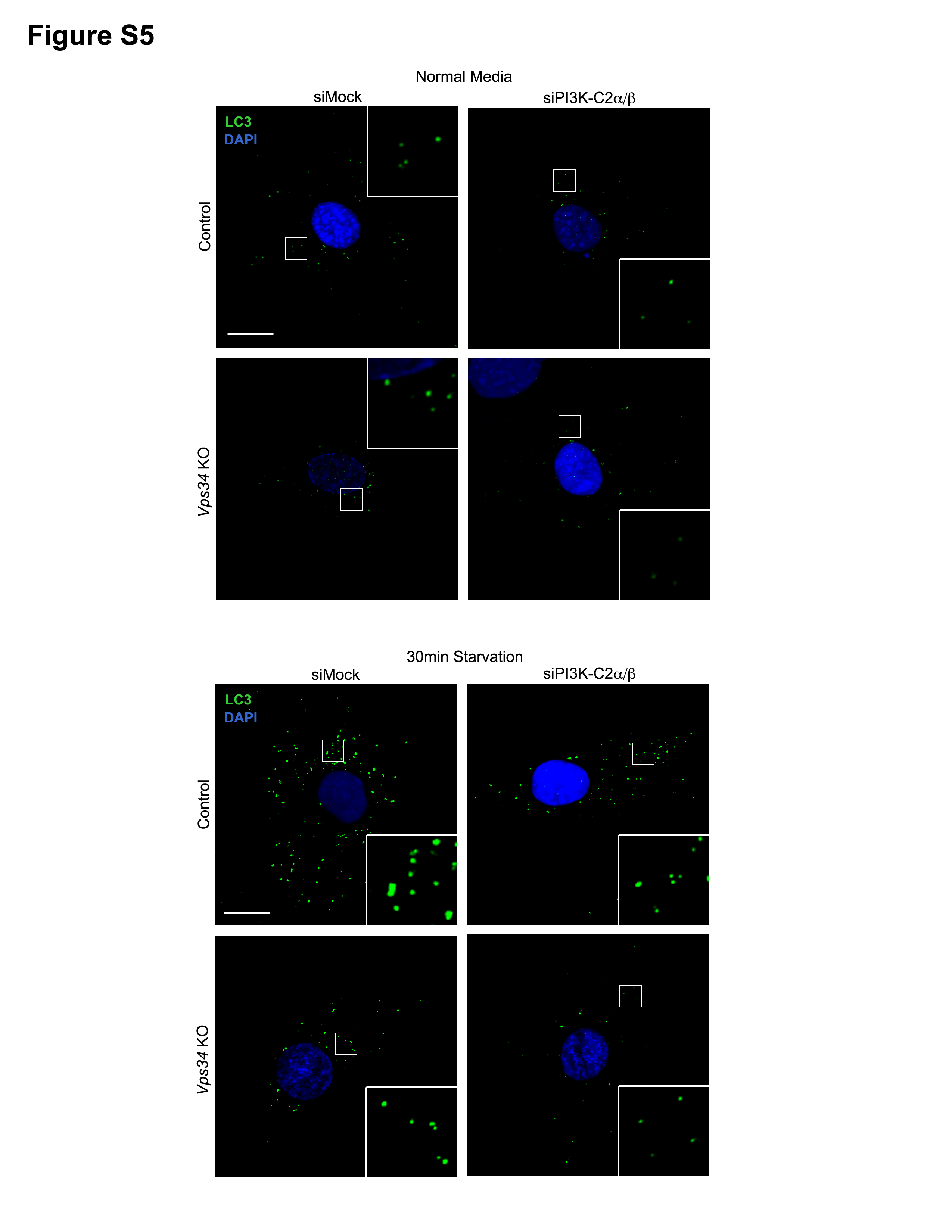

Supplement: Figure S5 — LC3 puncta formation during starvation-induced autophagy upon silencing class II PI3K in control and Vps34 KO MEFs. Control and Vps34 KO MEFs were transfected for 48 hrs with mock or PI3K-C2α/β siRNA, cultured in normal medium (N) or HBSS (St) for 30 min, fixed and immunostained. Representative confocal microscopy images showing endogenous LC3 (green) in cells cultured in normal media (top) or HBSS starvation conditions for 30min (bottom). DAPI is shown in blue. Scale bar: 10µm. (TIF) [file pone.0076405.s005.tif]
